# Recognition of Peptidoglycan Fragments by the Transpeptidase PBP4 From *Staphylococcus aureus*

**DOI:** 10.3389/fmicb.2018.03223

**Published:** 2019-01-18

**Authors:** Roberto Maya-Martinez, J. Andrew N. Alexander, Christian F. Otten, Isabel Ayala, Daniela Vollmer, Joe Gray, Catherine M. Bougault, Alister Burt, Cédric Laguri, Matthieu Fonvielle, Michel Arthur, Natalie C. J. Strynadka, Waldemar Vollmer, Jean-Pierre Simorre

**Affiliations:** ^1^University Grenoble Alpes, CNRS, CEA, IBS, Grenoble, France; ^2^Department of Biochemistry and Molecular Biology and Centre for Blood Research, The University of British Columbia, Vancouver, BC, Canada; ^3^Centre for Bacterial Cell Biology, Institute for Cell and Molecular Biosciences, Newcastle University, Newcastle upon Tyne, United Kingdom; ^4^Institute for Cell and Molecular Biosciences, Newcastle University, Newcastle upon Tyne, United Kingdom; ^5^Centre de Recherche des Cordeliers, LRMA, Equipe 12, Université Sorbone–Paris, Paris, France

**Keywords:** peptidoglycan, NMR, X-ray crystallography, penicillin-binding protein 4, *Staphylococcus aureus*, cyclic muropeptides

## Abstract

Peptidoglycan (PG) is an essential component of the cell envelope, maintaining bacterial cell shape and protecting it from bursting due to turgor pressure. The monoderm bacterium *Staphylococcus aureus* has a highly cross-linked PG, with ~90% of peptide stems participating in DD-cross-links and up to 15 peptide stems connected with each other. These cross-links are formed in transpeptidation reactions catalyzed by penicillin-binding proteins (PBPs) of classes A and B. Most *S. aureus* strains have three housekeeping PBPs with this function (PBP1, PBP2, and PBP3) but MRSA strains have acquired a third class B PBP, PBP2a, which is encoded by the *mecA* gene and required for the expression of high-level resistance to β-lactams. Another housekeeping PBP of *S. aureus* is PBP4, which belongs to the class C PBPs, and hence would be expected to have PG hydrolase (DD-carboxypeptidase or DD-endopeptidase) activity. However, previous works showed that, unexpectedly, PBP4 has transpeptidase activity that significantly contributes to both the high level of cross-linking in the PG of *S. aureus* and to the low level of β-lactam resistance in the absence of PBP2a. To gain insights into this unusual activity of PBP4, we studied by NMR spectroscopy its interaction *in vitro* with different substrates, including intact peptidoglycan, synthetic peptide stems, muropeptides, and long glycan chains with uncross-linked peptide stems. PBP4 showed no affinity for the complex, intact peptidoglycan or the smallest isolated peptide stems. Transpeptidase activity of PBP4 was verified with the disaccharide peptide subunits (muropeptides) *in vitro*, producing cyclic dimer and multimer products; these assays also showed a designed PBP4(S75C) nucleophile mutant to be inactive. Using this inactive but structurally highly similar variant, liquid-state NMR identified two interaction surfaces in close proximity to the central nucleophile position that can accommodate the potential donor and acceptor stems for the transpeptidation reaction. A PBP4:muropeptide model structure was built from these experimental restraints, which provides new mechanistic insights into *mecA* independent resistance to β-lactams in *S. aureus*.

## Introduction

Monoderm bacteria are surrounded by a cell wall containing a multi-layered peptidoglycan (PG) sacculus and secondary cell wall polymers such as teichoic acid and capsular polysaccharide (Silhavy et al., 2010). The PG is a mesh-like polymer that encases the cytoplasmic membrane to maintain the shape and rigidity of the cell. It is composed of glycan chains made of alternating β-1,4-linked *N*-acetylmuramic acid (MurNAc) and *N*-acetylglucosamine (GlcNAc) residues, which are connected by short peptides (Vollmer et al., 2008). PG precursor synthesis starts in the cytoplasm, where the UDP-MurNAc-peptide and UDP-GlcNAc precursors are assembled. The final PG precursor, lipid II, is then assembled at the inner leaflet of the cytoplasmic membrane, flipped across to the outer leaflet, and utilized by PG synthases to polymerize the glycan chains in processive glycosyltransferase (GTase) reactions and form the peptide cross-links by transpeptidation (TPase reactions) (Macheboeuf et al., 2006). The latter activity, catalyzed by penicillin-binding proteins (PBPs), is the target of β-lactam and glycopeptide antibiotics. In the context of the increasing problem of antimicrobial drug resistance and the role of PG fragments in the innate immune response, it is crucial to get more information on the interactions between proteins and cell wall components such as PG (Zapun et al., 2008; Sung et al., 2009). Some PG-interacting proteins are able to bind antibiotics or soluble PG fragments with an affinity that is amenable to structural studies using X-ray crystallography (Sobhanifar et al., 2013). For example, the structure of the TPase PBP2x from *Streptococcus pneumoniae* has been determined by crystallography. A hypothetical model of the possible complex with a large peptidoglycan fragment has been proposed based on structures of non-covalent and covalent PBP complexes with β-lactam antibiotics (Bernardo-García et al., 2018). Liquid-state NMR has also been used to determine the structure of complexes with lower affinity (Lehotzky et al., 2010). However, interaction studies involving large fragments or the entire peptidoglycan polymer are in most cases not amenable to liquid-state NMR. Furthermore, the peptidoglycan sacculus is a large (10^9^ Da), dynamic, and heterogeneous structure, which hampers structural investigations by electron microscopy and X-ray crystallography. Solid-state NMR has emerged as a promising method to characterize peptidoglycan structure and dynamics (Kern et al., 2010; Romaniuk and Cegelski, 2015). Solid-state NMR can be used with any sample whose molecules re-orient on a time scale that is much slower than the ms-range time-scale of the NMR experiment. Thus, solid-state NMR can be applied to hydrated insoluble cell walls or hydrated intact cell samples, with the advantage of an improved spectral resolution resulting from the local dynamics present in the hydrated state (Gang et al., 1997; Kern et al., 2010). In this context, solid-state NMR has been used to measure structural constraints on a complex formed between the LD-transpeptidase from *Bacillus subtilis* Ldt_Bs_ and intact peptidoglycan sacculi (Schanda et al., 2014).

Infection with methicillin-resistant *Staphylococcus aureus* (MRSA) results in diverse clinical manifestations, ranging from minor skin infections to life-threatening bacteremia and pneumonia. *S. aureus* has one monofunctional GTase and four PBPs, of which PBP2, the sole bifunctional class A PBP, is responsible for the majority of PG synthesis (Pinho and Errington, 2004; Sauvage et al., 2008). PBP2 is essential in *S. aureus* strains susceptible to methicillin, but its TPase activity can be replaced by that of an acquired and unusual class B PBP, PBP2a, when cells are grown in the presence of methicillin (Pinho et al., 2001). Of the two other class B PBPs, the essential PBP1 plays a role in cell division and separation, whereas the function of the non-essential PBP3 is still vague (Pinho et al., 2000; Pereira et al., 2007). PBP4 is the only class C PBP present in *S. aureus*. Members of this class usually exhibit DD-carboxypeptidase (DD-CPase) or endopeptidase (DD-EPase) activity. PBP4 from *S. aureus* is unique within the class C PBPs, as it was shown *in vivo* and *in vitro* to possess DD-TPase activity in addition to DD-CPase activity, leading to a highly cross-linked PG (Wyke et al., 1981; Loskill et al., 2014; Srisuknimit et al., 2017). PBP4 does not appear to work on nascent PG, but catalyzes further cross-linking reactions in polymeric PG (Atilano et al., 2010).

To perform its transpeptidase activity, PBP4 initiates a nucleophilic attack by the hydroxyl group of the catalytic Ser75 residue on the terminal D-Ala-D-Ala amide bond of the peptidoglycan stem peptide. The C-terminal D-Ala is subsequently released from the peptide and an acylenzyme intermediate forms. Enzyme deacylation follows when the terminal amino group of the glycine bridge of a second peptide stem acts as an acyl acceptor, resulting in a peptide cross-link between two adjacent peptidoglycan stems. The CPase activity follows a similar reaction scheme, except that the acceptor is a water molecule, yielding a tetrapeptide stem after enzyme deacylation. The β-lactam ring of methicillin and other antibiotics of the β-lactam family can act as mimics of the D-Ala-D-Ala extremity of the acyl-donor peptide stem. However, unlike the natural substrate, the β-lactam–PBP acylenzyme is very stable and results in long-term inactivation of the PBPs. Different β-lactam–PBP acylenzymes have been structurally characterized at atomic resolution but muropeptide- or peptidoglycan-PBP adducts have thus far only been modeled due to the intractable nature of crystallizing these larger substrate complexes (Bernardo-García et al., 2018). Some methicillin-resistant strains of *S. aureus* carry mutations in either the promotor region or the *pbp4* gene itself, leading to an increased expression of PBP4 and/or increased PG cross-linkage but without significantly altering β-lactam binding (Chatterjee et al., 2017; Hamilton et al., 2017).

Rational modification of the β-lactam scaffold to potentially overcome PBP4-mediated resistance would therefore require additional structural information on the PG fragment-PBP4 adducts. To obtain such data, we studied here the interaction of PBP4 with different natural substrates or substrate analogs. We characterized the activity of PBP4 *in vitro* and measured the interaction between PBP4 and different PG fragments by X-ray crystallography, liquid- and solid-state NMR. From the NMR data obtained we built a structural model of a peptidoglycan fragment-PBP4 complex that may help explain the unusual DD-TPase activity of this PBP.

## Materials and Methods

### Preparation of Peptidoglycan and Muropeptides

*S. aureus* strain SH1000 (wild type) was a generous gift of S. J. Foster (University of Sheffield). *S. aureus* cells were grown in 1.5 L of Tryptic Soy Broth (TSB) until an OD_600_ of 0.8–0.9 (Bui et al., 2012; Figueiredo et al., 2012). Cells were subsequently cooled down at 4°C and harvested by centrifugation at 10,000 × g for 20 min. Bacterial cell walls were purified from the cell pellets according to a published protocol for *Streptococcus pneumoniae* (Bui et al., 2012). To remove wall teichoic acids, 10 mg of cell wall was treated with 48% hydrofluoric acid at 4°C for 48 h. PG (~5 mg) was recovered by centrifugation at 264,000 × g and 4°C for 45 min. The PG pellet was washed with water and resuspended in 1 mL of buffer containing 0.02% sodium azide for storage at 4°C. Uniformly ^13^C,^15^N-labeled peptidoglycan SH1000 samples were obtained by growing *S. aureus* cells in a M9 medium containing 4 g/L of ^13^C-glucose and 1 g/L of ^15^NH_4_Cl.

To generate muropeptides, 150 μL of the *S. aureus* SH1000 stock suspension of PG was incubated overnight at 37°C with 50 μL of 320 mM sodium phosphate, pH 4.8 and 10 μL of 1 mg/mL cellosyl (Höchst AG, Frankfurt, Germany). The mixture was boiled at 100°C for 10 min to inactivate the enzyme, the sample was centrifuged at 10,000 × g for 20 min, and the supernatant containing the muropeptides was collected. The muropeptide solution was stored at 2–8°C. To generate the soluble PG glycan chains with monomeric peptides the PG was digested with recombinant lysostaphin from *Staphylococcus simulans* (Sigma-Aldrich). In a typical preparation, 20 mL of a 5 mg/mL suspension of unlabeled or ^13^C,^15^N-labeled PG in 5 mM sodium phosphate at pH 7.0 was incubated under stirring at 37°C for 24 h with 2 mg of lysostaphin (final concentration 100 μg/mL). Lysostaphin was inactivated and precipitated by placing the mixture at 100°C for 10 min. The mixture was centrifuged at 10,000 × g for 30 min and the supernatant containing the PG fragments was recovered and stored at −20°C.

Soluble fragments were dialyzed against water. Before aliquoting the samples or before lyophilization, the concentration of the stock solution was estimated by liquid-state NMR using the Eretic pulse sequence (Frank et al., 2014) and a reference 1 mM sucrose sample in 90%:10% H_2_O:D_2_O.

### Synthesis of Branched Lactoyl Peptides

Fmoc-D-Glu-NHTrt and D-LacO^t^Bu were synthetized as previously described (see Supporting Information in Ngadjeua et al., 2018). Orthogonal Fmoc and 1-(4,4-dimethyl-2,6-dioxocyclohexylidene)-3-methylbutyl (ivDde) protecting groups were used for sequential assembly of the branched peptide stem (see Ngadjeua et al., 2018 for similar peptides containing a D-iAsn bridge). First, the main peptide stem (D-Lac-L-Ala-D-iGln-L-Lys-D-Ala_1or2_) was elongated from the Wang-D-Ala resin by successive coupling reactions with Fmoc-protected amino-acids. Second, the ε-NH_2_ group of L-Lys was deprotected by hydrazinolysis, and Fmoc-Gly and 2 Fmoc-Gly-Gly were successively introduced to build the pentaglycine-bridge in three steps (**Figure 2A**). Final deprotection of acid-labile Fmoc and Trt protecting groups and cleavage from the resin was achieved under gentle stirring at room temperature with 2 mL of a TFA solution containing DCM, TIPS, and water (80:20:5:5 v:v:v:v). The solution was filtered to remove the resin, which was washed with 1 mL of the TFA solution. The TFA solutions were pooled and evaporated under reduced pressure before solvent extraction and purification by *rp*HPLC (Supplementary Information). 11 mg (14.2 μmol) and 4 mg (4.7 μmol) of pure tetrapeptide and pentapeptide, respectively, were isolated. The purity of each sample was analyzed by HPLC and their chemical structure characterized by mass spectrometry (Supplementary Figure S2) and NMR (Supplementary Figure S1 and Supplementary Table 1).

### Expression and Purification of *S. aureus* PBP4 and PBP4(S75C)

Unlabeled recombinant native PBP4 (residues 21-383) was expressed as previously published (Hamilton et al., 2017). Unlabeled PBP4(S75C) was expressed, purified and treated with thrombin to remove the His-tag as previously reported for the native enzyme, except that 5 mM TCEP was included in all buffers used during purification (Alexander et al., 2018). The expression and purification protocol was adapted for the production of ^13^C,^15^N-labeled PBP4 and PBP4(S75C) samples for NMR studies. To maintain good protein yields, thrombin cleavage of the GSSHHHHHHSSGLVPRGSHM N-terminal His-tag was not performed. Plasmid carrying the PBP4 or PBP4(S75C) gene was transformed into *E. coli* BL21(DE3). Freshly transformed bacteria were sequentially adapted over 1 day from LB to minimal M9 medium (37 mM Na_2_HPO_4_, 22 mM KH_2_PO_4_, 8.5 mM NaCl, 1 g L^−1^ ^15^NH_4_Cl, 2 g L^−1^ D-glucose-^13^C_6_ for ^13^C,^15^N-labeled proteins or D-glucose for ^15^N-only labeled proteins, 1 mM MgSO_4_, 0.1 mM CaCl_2_, 0.1 mM MnCl_2_, 50 μM ZnSO_4_, 50 μM FeCl_3_, 1 mg pyridoxine, 1 mg biotin, 1 mg hemicalcium salt of panthothenic acid, 1 mg folic acid, 1 mg choline chloride, 1 mg niacinamide, 0.1 mg riboflavin, 5 mg thiamine). M9 preculture grown overnight at 37°C with OD_600_ ~2–2.5 was used to inoculate 1 L of M9 culture medium in a 1:10 ratio. The latter culture was grown at 37°C until OD_600_ = 1. Expression was then induced for 3 h at 37°C with 1 mM IPTG. After harvesting the cells by centrifugation at 6,000 × g and 4°C for 20 min, the pellet was resuspended in 20 mL of lysis buffer (25 mM Tris-HCl, 500 mM NaCl, 20 mM imidazole at pH 7.5, which contained in addition 2 mM β-mercaptoethanol in the case of PBP4(S75C)). One tablet of cOmplete EDTA-free (Roche), 10 mg of lysozyme and RNase/DNase were added, and cells were disrupted by sonication. The lysate was clarified by centrifugation at 46,000 x g during 45 min at 4°C and cell debris were discarded. The supernatant was loaded onto a HisTrap™ (GE Healthcare) chromatography column. A wash was performed with 5 column-volumes of Buffer A (25 mM Tris-HCl, 1 M NaCl, 20 mM imidazole at pH 7.5). The protein was eluted with a 10–100% linear gradient of Buffer B (25 mM Tris-HCl, 500 mM NaCl, 500 mM imidazole at pH 7.5). Fractions containing the protein were pooled and concentrated and then loaded to a Superdex200 (16/60) column pre-equilibrated with the NMR buffer (100 mM potassium phosphate, 150 mM KCl at pH 7.0). Fractions containing the pure protein were pooled and concentrated for the requirements of NMR studies. For the production of the ^13^C,^15^N,^2^H-labeled PBP4 sample, two additional pre-cultures were performed before the 1L-culture for the sequential adaptation of cells to a 50%H_2_O:50%D_2_O M9 medium and 100% D_2_O M9 medium. The 1L-culture was furthermore achieved in 100% D_2_O with D-Glucose-^13^${\text{C}}_{6}^{2}$H_7_.

### Pull-Down Experiments

Stored sacculi were resuspended, washed twice with water and twice with the interaction buffer, 100 mM potassium phosphate (pH 7.0), and isolated by centrifugation for 10 min at 16,000 × g. The PBP4 protein sample was prepared as described previously and the buffer was exchanged on a NAP™-5 desalting column (GE-Healthcare) pre-equilibrated with the interaction buffer, and the protein concentration was adjusted to 20 μM. After resuspension in the interaction buffer, 100 μL of sacculi solution was incubated with 100 μL of 20-μM PBP4 at 4°C overnight. The supernatant was discarded and kept for analysis by SDS-PAGE. Sacculi were washed 3 times with 100 μL of interaction buffer and finally resuspended in Laemmli buffer for SDS-PAGE analysis.

### PBP4 Activity Assays With Muropeptides or With PG From *S. aureus* SH1000

Assays were carried out in a final volume of 50 μL containing 10 mM Tris/HCl at pH 7.5, 10 mM MgCl_2_, 0.1% Triton X-100, and 10 μM PBP4 or PBP4(S75C). A ~5 mg mL^−1^ suspension of PG or 25 μL of a ~5 mg mL^−1^ solution of muropeptides were added and the reaction mixture was incubated at 37°C overnight. The enzymatic reaction was stopped by boiling the samples for 10 min. Muropeptides were reduced and analyzed by HPLC as described (Boneca et al., 1997).

### PBP4(S75C) Activity Assays With Imipenem and Native Mass Spectrometry

Purified PBP4(S75C) at 1.2 mg mL^−1^ was incubated at ~23°C for 30 min in buffer C (20 mM MES pH 6, 300 mM NaCl, 5 mM Tris(2-carboxyethyl)phosphine (TCEP)) with or without 1.25 mM imipenem prior to being frozen at −20°C. For mass spectrometry analysis, samples were thawed and diluted 500 times in 5% acetonitrile and 0.1% formic acid. 5 μL of each sample was injected onto a 5-mm C4 column connected to a Waters Xevo GS-2 QTof mass spectrometer via a NanoAquity UPLC system. Samples were eluted in a 2-min gradient from 5 to 100% acetonitrile at a flow rate of 20 μL min^−1^. Mass spectra (Supplementary Figure S3) were summed and peak masses deconvoluted using Waters' MassLynx software (V4.1).

### X-Ray Crystallography of PBP4(S75C)

PBP4(S75C) was crystallized using the sitting drop vapor diffusion method with streak seeding. Protein at 25–35 mg mL^−1^ was added to precipitant solution (8 mM zinc chloride, 80 mM sodium acetate pH 5, 100 mM sodium fluoride, and 16% polyethylene glycol 6000) in a 1:1 (v/v) ratio and incubated at 23°C. Immediately after mixing the protein and precipitant solution, the drops were streak-seeded with a housecat whisker that had been swished through a drop containing PBP4(S75C) crystals. Prior to harvesting, the PBP4(S75C) crystals were soaked in 1 mM ampicillin and 8 mM HO-D-Lac-L-Ala-D-iGln-[L-Lys(Gly)_5_]-D-Ala-D-Ala-COOH pentapeptide for 40–60 min before glycerol was added to 15% (v/v). Crystals were then promptly harvested and stored in liquid nitrogen.

Data collection was performed under cryogenic temperatures (100 K) at the Advanced Light Source synchrotron (U.C. Berkeley) on beamline 5.0.2. Data from one crystal that diffracted to 1.9 Å resolution were processed using Xia2 (Winter et al., 2013) and XDS (Kabsch, 2010) with a space group of C121 and merged with Aimless in the CCP4 software package (Winn et al., 2011). Phaser (McCoy et al., 2007) was employed to solve the structure by molecular replacement using PDB ID 5TXI as the starting model. The Phenix suite of programs was used for model generation and refinement (Adams et al., 2010). Briefly, AutoBuild was used with several iterative rounds of manual manipulation of the model with *Coot* (Emsley et al., 2010) followed by refinement with Phenix.refine with TLS being used in the later stages of refinement. Supplementary Figure S4 was generated using PyMOL (The PyMOL Molecular Graphics System, Version 2.1.1 Schrödinger, LLC). The final structure was deposited into the PDB under the accession code 6DZ8.

### NMR Resonance Assignment

Data for ^1^H- and ^13^C-NMR resonance assignment of synthetic tetra- and pentapeptide were recorded at 20°C on a 2 mg mL^−1^ solution of the peptide in 50 mM potassium phosphate buffer at pH 6.5 with 10% D_2_O on a Bruker 600-MHz spectrometer equipped with an Avance IIIHD console and a cryogenically cooled triple-resonance probe. Collected experiments on both peptides included 1D ^1^H, 2D-TOCSY and 2D-NOESY with excitation sculpting for water suppression and sensitivity-enhanced ^13^C-HSQC.

Data for ^13^C,^15^N-labeled peptidoglycan fragments from *S. aureus* strain SH1000 obtained by lysostaphin digestion were recorded at 20°C on a 2 mg mL^−1^ solution in 50 mM potassium phosphate buffer at pH 6.5 with 10% D_2_O on a Bruker 850-MHz spectrometer equipped with an Avance IIIHD console and a cryogenically cooled triple-resonance probe. 2D ^13^C-HSQC and ^15^N-BEST-TROSY experiments served as starting points for assignments. 3D BEST-HNCACB, BEST-HNcoCACB, BEST-HNCO, HccoNH, hCcoNH, hNcacoNH experiments were collected for the assignment of resonances to nuclei of peptide stems ^1^H, ^13^C and ^15^N (Favier and Brutscher, 2011). These experiments were complemented with ^15^N- and ^13^C-NOESY-HSQC. A 2D hCcH-TOCSY dataset was collected for the assignment of carbohydrate resonances.

Data for ^1^H-, ^13^C- and ^15^N-NMR backbone resonance assignment of wild-type PBP4 were recorded at 25°C on an 800 μM ^13^C,^15^N,^2^H-labeled PBP4 sample in 100 mM potassium phosphate buffer containing 300 mM KCl and 5% D_2_O at pH 7.5 on Bruker spectrometers with ^1^H Larmor frequencies ranging from 700 to 950 MHz equipped with an Avance IIIHD console and a cryogenically cooled triple-resonance probe. The superimposition of the ^15^N-BEST-TROSY of this sample and an equivalent ^13^C,^15^N-only labeled sample proved the H/D back-exchange to be essentially complete in the conditions used to purify the protein. Collected experiments included a ^15^N-BEST-TROSY, a BEST-HNCACB/BEST-HNcoCACB pair, a BEST-HNCA/BEST-HNcoCA pair, a BEST-HNCO/BEST-HNcaCO pair, and a ^15^N-NOESY-HSQC. Deuteration of this 384-amino acid protein construct was instrumental for magnetization transfer through scalar couplings in 3D experiments. All NMR data were processed with Topspin 3.5 (Bruker) and analyzed with CcpNmr (Vranken et al., 2005) for resonance assignment. Assignments were 100% complete for synthetic peptides and peptidoglycan fragments. For PBP4 unambiguous resonance assignment was obtained for 65.5, 67.7, 69.0, and 67.0% of the backbone amide nuclei, carbonyl carbons, α-carbons, and β-carbons, respectively. Close to 13% of the amide resonances remained undetected at pH 7.5 and on the pH-stability range of the protein. The corresponding residues are probably in dynamic part of the protein in solution or their backbone amide is largely accessible to the solvent. Among the amide resonances, 25% were detected but remained unambiguously assigned due to coherence transfer issues in the 3D experiments and signal overlap. Assignments of resonances on wild-type PBP4 were transferred to PBP4(S75C) upon superimposition of ^15^N-BEST-TROSY experiments recorded in the same conditions.

### NMR Titration Experiments

Interaction studies with ^15^N-labeled PBP4(S75C) and different substrates were monitored by superimposition of ^15^N-BEST-TROSY spectra at 25°C for different substrate-to-protein ratio. Synthetic peptide stems and peptidoglycan fragments were extensively dialyzed against water using a Spectra/PorTM dialysis membrane with a 100–500 Da cutoff (Spectrum Laboratories, Inc.) and lyophilized before preparation of a few-mM stock solution in the protein buffer, with 50 mM potassium phosphate at pH 6.5. A 326-μM solution of PBP4(S75C) was titrated with 2, 8, 22, and 52 molar equivalents of tetrapeptide. A 150-μM solution of PBP4(S75C) was titrated with an estimated 2, 10, 25, 50, 100, and 180 molar equivalents of muropeptides from *S. aureus* strain SH1000. A 150-μM solution of PBP4(S75C) was titrated with estimated 2, 10, and 16 molar equivalents of peptidoglycan fragments obtained by lysostaphin digestion. A 150-μM solution of PBP4(S75C) was incubated with 1.2 molar equivalents of imipenem and showed significant chemical shift changes for some of the resonances. Addition of another 1.2 molar equivalents of imipenem did not yield further chemical shift changes. CcpNmr was used to monitor protein chemical shift perturbations (CSP) for every assigned amide resonance by superimposition of the ^15^N-BEST-TROSY spectra. CSPs (Δδ) were calculated on a per-residue basis for the highest substrate-to-protein ratio as follows:

$$\begin{array}{l} \Delta \delta =\sqrt{{\left(\delta_{PBP4^{S75C}:sub}^{1_{H}}-\delta_{PBP4^{S75C}}^{1_{H}}\right)}^{2}+{\left[\frac{\gamma_{N}}{\gamma_{H}}\left(\delta_{PBP4^{S75C}:sub}^{15_{N}}-\delta_{PBP4^{S75C}}^{15_{N}}\right)\right]}^{2}}\text{,} \end{array}$$

where γ_*N*_ and γ_*H*_ are the ^1^H and ^15^N gyromagnetic ratios, respectively, and ${\delta^{X}}_{PBP4\left(S75C\right):sub}$, and ${\delta^{X}}_{PBP4\left(S75C\right)}$ are the protein resonance chemical shift value in the presence and absence, respectively, of the substrate at the highest ratio.

### NMR-Data Driven Docking

Models of PBP4 in complex with muropeptides were built with the version HADDOCK2.2 of “The HADDOCK web server for data-driven biomolecular docking” (de Vries et al., 2010). As starting structures, we used the X-ray crystallography structure of PBP4 (PDB ID 6C39) and a GlcNAc-MurNAc-L-Ala-D-iGln-[L-Lys(Gly)_5_]-D-Ala muropeptide structure generated in-house using CNS (Schanda et al., 2014). The PBP4 and two muropeptide structures were then docked within HADDOCK. All atoms of the muropeptides were considered as passive Ambiguous Restraints (AIR). Residues of PBP4 that showed chemical shift perturbations above the threshold in Supplementary Figure S6 were considered as active AIRs. Calculations were performed with 2,000 structures during the HADDOCK rigid body energy minimization, 400 structures during the refinement, and 200 structures during the refinement in explicit water. Structure scores were calculated from the weighted energy average, HADDOCK score = E_vdW_ + 0.2 E_Electrostatics_ + 0.1 E_Air_ + E_desolvation_. The output model structures were sorted with the HADDOCK built-in clustering tool using the Fraction of Common Contacts (FCC) method (Rodrigues et al., 2012) with a 0.61-Å cutoff and a minimum of 4 structures per cluster. To improve the convergence during the HADDOCK run, a 2.5-Å unambiguous restraint was introduced between the oxygen of the catalytic serine S75 of PBP4 and the carbonyl carbon of D-Ala^4^ of one muropeptide stem, as well as between the oxygen of the catalytic serine S75 of PBP4 and the amine nitrogen of the bridging Gly5 of another muropeptide stem. After on-line FCC clustering of the solutions, the two clusters with the best HADDOCK scores and lower energy were analyzed in more detail within PyMOL.

## Results

### PBP4 Does Not Interact With High-Molecular Weight PG

**Figure 1:**
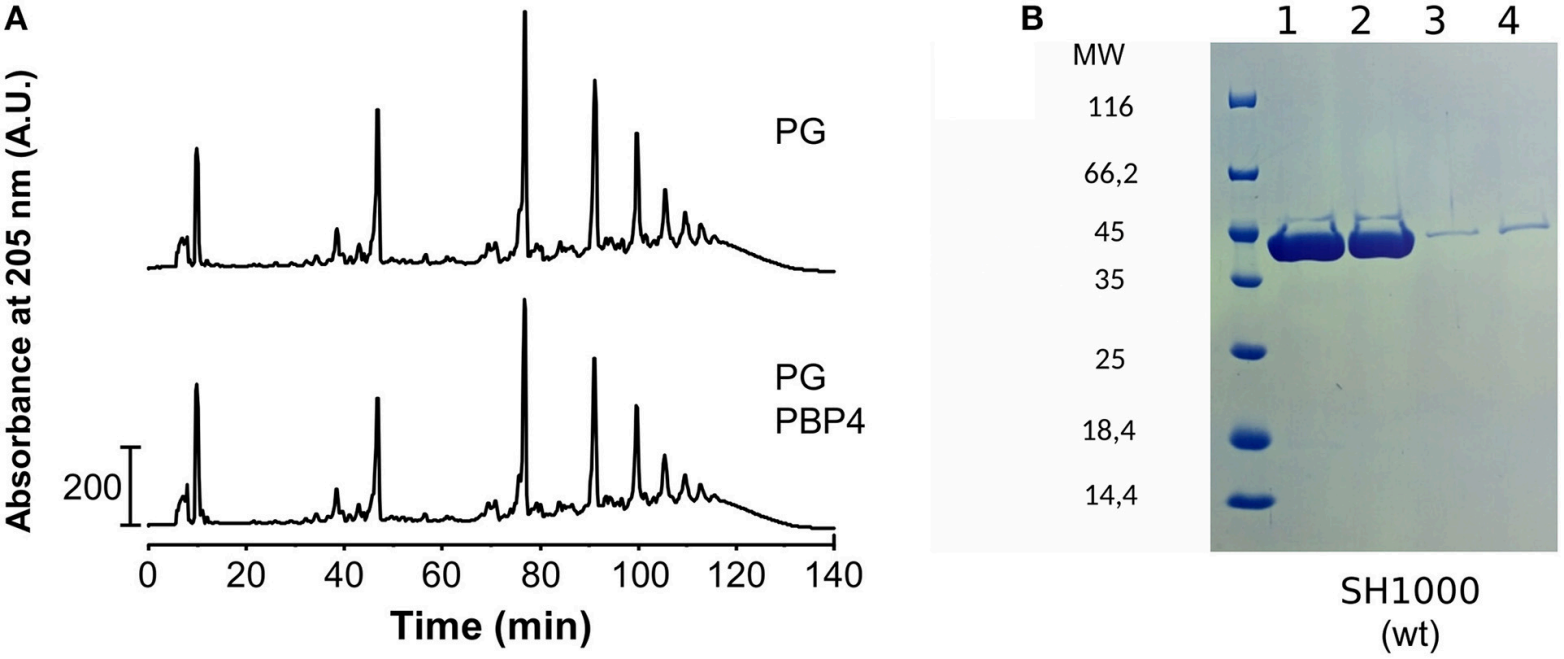
PBP4 does not modify or interact with purified PG. **(A)** PG from *S. aureus* SH1000 was incubated with PBP4, followed by preparation and HPLC analysis of muropeptides. The muropeptide profile of the sample with PBP4 (bottom chromatogram) showed no significant difference to the control sample without PBP4 (top). This shows that PG is not a substrate for PBP4. **(B)** Interaction of PBP4 with peptidoglycan sacculi using pull-down experiments. Pull-down experiments were performed with purified PG from *S. aureus* strain SH1000. Samples were analyzed by SDS-PAGE stained by Coomassie Blue. PBP4 was present in the supernatant fractions (lanes 1 and 2) and largely absent in the PG fractions (lanes 3 and 4) showing that PBP4 does not interact with PG.

As PBP4 contributes to the high degree of cross-linking in the PG of *S. aureus*, we first considered the mature peptidoglycan of *S. aureus* as a substrate for PBP4 DD-TPase activity. We thus incubated the enzyme with PG from *S. aureus* strain SH1000, digested the resulting PG with cellosyl muramidase, and analyzed the obtained muropeptide profile by HPLC. PBP4 did not alter the muropeptide profile, indicating that high-molecular weight PG is not a substrate for the transpeptidase reaction (Figure 1A). However, it is possible that high-molecular weight PG is recognized and coordinated by the enzyme in an accessory role. To test this, we isolated PG from *S. aureus* cells and assayed for interaction with PBP4 by a pull-down experiment. PBP4 was not pulled down with PG, showing that the enzyme does not have a high affinity for PG (Figure 1B). This result also precluded being able to perform binding experiments by solid-state NMR spectroscopy and, therefore, we turned to liquid-state NMR spectroscopy approaches to probe for interactions between PBP4 and soluble peptidoglycan fragments.

### Preparation of Different Soluble Peptidoglycan Fragments

**Figure 2:**
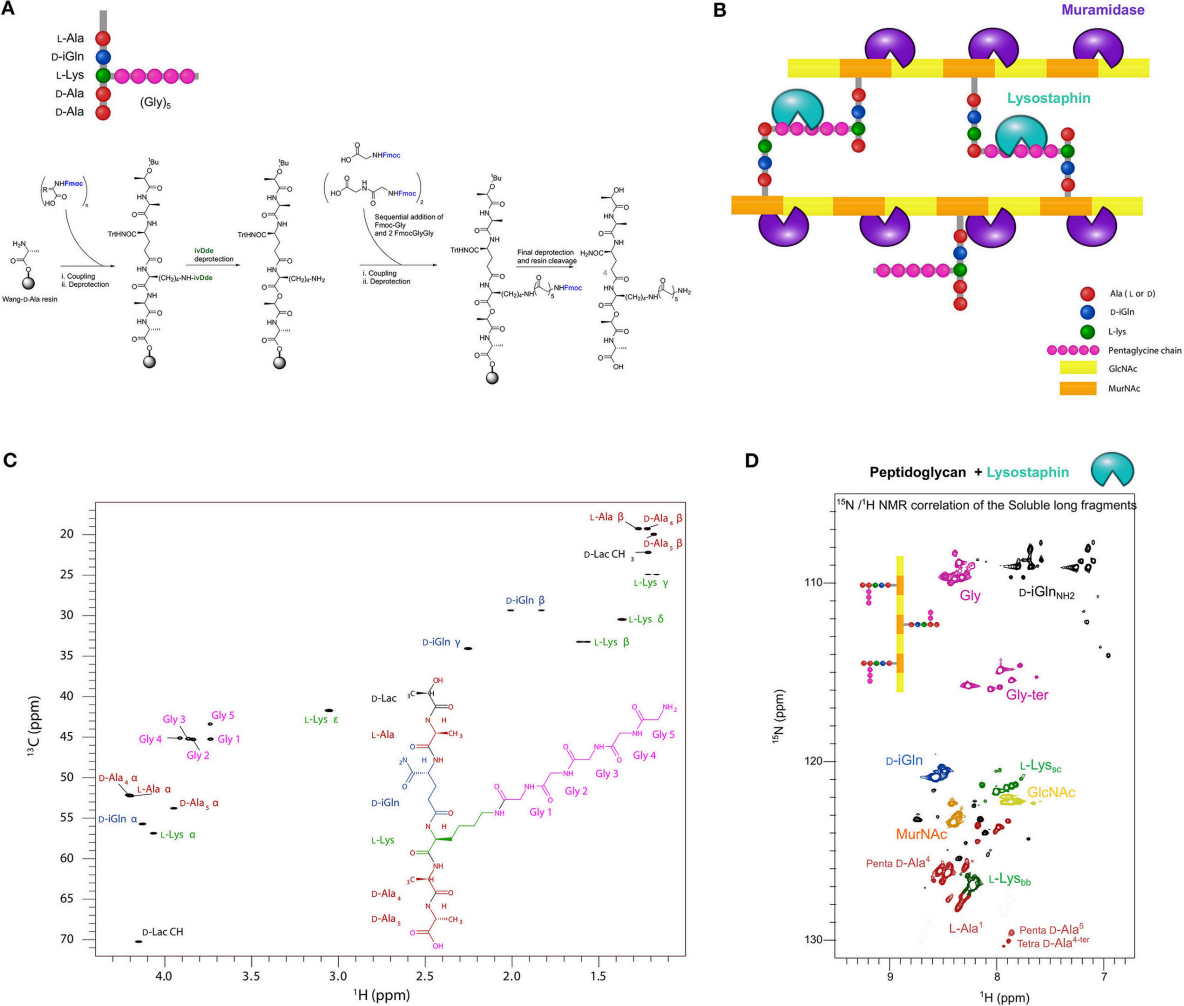
Preparation and NMR spectroscopy of PG fragments **(A)** Scheme of the synthetic route to branched lactoyl-peptides. Wang-D-Ala resin was obtained by Fmoc deprotection of the commercially available functionalized solid-phase support for peptide synthesis. Protecting groups: Fmoc, fluorenylmethoxycarbonyl; Trt, trityl; IvDde, 1-(4,4-dimethyl-2,6-dioxocyclohexylidene)-3-methylbutyl. **(B)** Scheme of the PG structure showing the cleavage sites of the muramidase cellosyl (purple) and the endopeptidase lysostaphin (green). **(C)** ^1^H,^13^C-correlation NMR spectrum (^13^C-HSQC) collected at 20°C on a 2.3 mM sample of HO-D-Lac-L-Ala-D-iGln-L-Lys(Gly)_5_-D-Ala-D-Ala-COOH pentapeptide in 50 mM potassium phosphate buffer, pH 6.5 containing 10% D_2_O. **(D)** ^1^H,^15^N-correlation NMR spectrum (^15^N-HSQC) of a ~500 μM solution of PG fragments obtained by digestion with lysostaphin of PG from *S. aureus* SH1000 grown in a ^13^C,^15^N-labeled medium. Resonances are color-coded with residue-type. Bb and sc indices stand for backbone and side-chain, respectively. The percentage of peptides with one, two or more Gly residues attached to the ε- amino group of Lys (after processing with lysostaphin) was ~6% of (1 Gly), ~51% of (2 Gly), and ~42% of (more than 2 Gly), suggesting a preferred cleavage of the pentaglycine bridge between the Gly residues at positions 2 and 3.

In order to proceed we aimed to prepare different soluble peptidoglycan fragments to analyze enzyme/substrate complexes by X-ray crystallography or liquid-state NMR spectroscopy. As potential DD-TPase, DD-CPase and DD-EPase activities of PBP4 target the peptides in PG, we first considered chemical synthesis of defined tetrapeptide and pentapeptide stems. To this end, we synthesized two branched lactoyl-peptides, HO-D-Lac-L-Ala-D-iGln-L-Lys(Gly)_5_-D-Ala-COOH tetrapeptide and HO-D-Lac-L-Ala-D-iGln-[L-Lys(Gly)_5_]-D-Ala-D-Ala-COOH pentapeptide, which both resemble the native peptides in the PG of *S. aureus*, by divergent Fmoc solid-phase peptide synthesis using orthogonal protecting groups (see Methods) in 24 and 8% yield, respectively (Figure 2A). Milligram quantities were obtained for each of the tetrapeptide and pentapeptide products, following HPLC purification, solvent exchange against water and lyophilization. We also prepared alternative possible substrates for PBP4 by digesting PG from *S. aureus* by different hydrolases (Figure 2B). For this, PG was prepared from *S. aureus* grown either in unlabeled growth media or in growth media for ^13^C,^15^N-isotopic labeling. The PG was digested overnight with the cellosyl muramidase or the lysostaphin endopeptidase. Cellosyl generates a mixture of cross-linked and un-crosslinked disaccharide peptide subunits (muropeptides); lysostaphin generates glycan chains bearing un-crosslinked peptides. After heat inactivation of the hydrolases, the PG fragments were extensively dialyzed against water using a 500-kDa cut-off membrane, and lyophilized. In each case we recovered a few mg of a white powder of soluble peptidoglycan fragment mixture from approximately 10 mg of PG. Synthetic peptides and ^13^C,^15^N-labeled soluble fragments prepared from PG were analyzed by liquid-state NMR spectroscopy using different homonuclear or heteronuclear experiments. Figure 2C shows the resonance assignments for the ^1^H,^13^C-correlation spectrum of the synthetic pentapeptide, confirming its chemical structure (see Supplementary Figure S1A for tetrapeptide spectra, Supplementary Figure S2 for HPLC and MS characterization and Supplementary Table 1 for resonance assignments on both peptides). Figure 2D shows the ^1^H,^15^N-correlation spectrum of soluble PG fragments obtained by digestion with lysostaphin (see Supplementary Figure S1B for ^1^H,^13^C-correlations). The intensities and line widths of the resonances suggested that the generated fragments behave mainly as monomers in solution. Resonance assignments through 3D heteronuclear experiments aided the characterization of fragments of different chemical structures within the mixture (Figure 2D). The amide resonances of terminal residues of a peptide chain could be clearly distinguished from the resonances derived from amino acids present within the peptide. This allowed us to identify the terminal D-Ala^4^ and D-Ala^5^ in the tetrapeptides and pentapeptides, respectively, and to quantify each species in the mixture. Similarly, terminal glycines with a free carboxylic acid and penultimate glycines with a free amine (resulting from the hydrolysis of a Gly-Gly peptide bond by lysostaphin) produce a ^15^N-chemical shift ranging from 114 to 117 ppm, while amide resonances from internal glycine residues show ^15^N chemical shifts ranging from 107 to 111 ppm (in pink in Figure 2D). Quantifying the signals from these two sets of Gly resonances revealed that lysostaphin cleaved the glycine bridge to leave mainly 2 to 3 Gly residues at the Lys, showing that lysostaphin predominantly cleaves the pentaglycine-bridge at these positions. This observation is in agreement with results obtained from lysostaphin digestion of the cell wall-anchored MalE-Cws protein, which yielded a major soluble protein fraction with the addition of three Gly from the PG pentaglycine bridge at its C-terminus as shown by mass spectrometry (Schneewind et al., 1995).

### PBP4 Produces Cyclic Muropeptides by TPase Reactions

**Figure 3:**
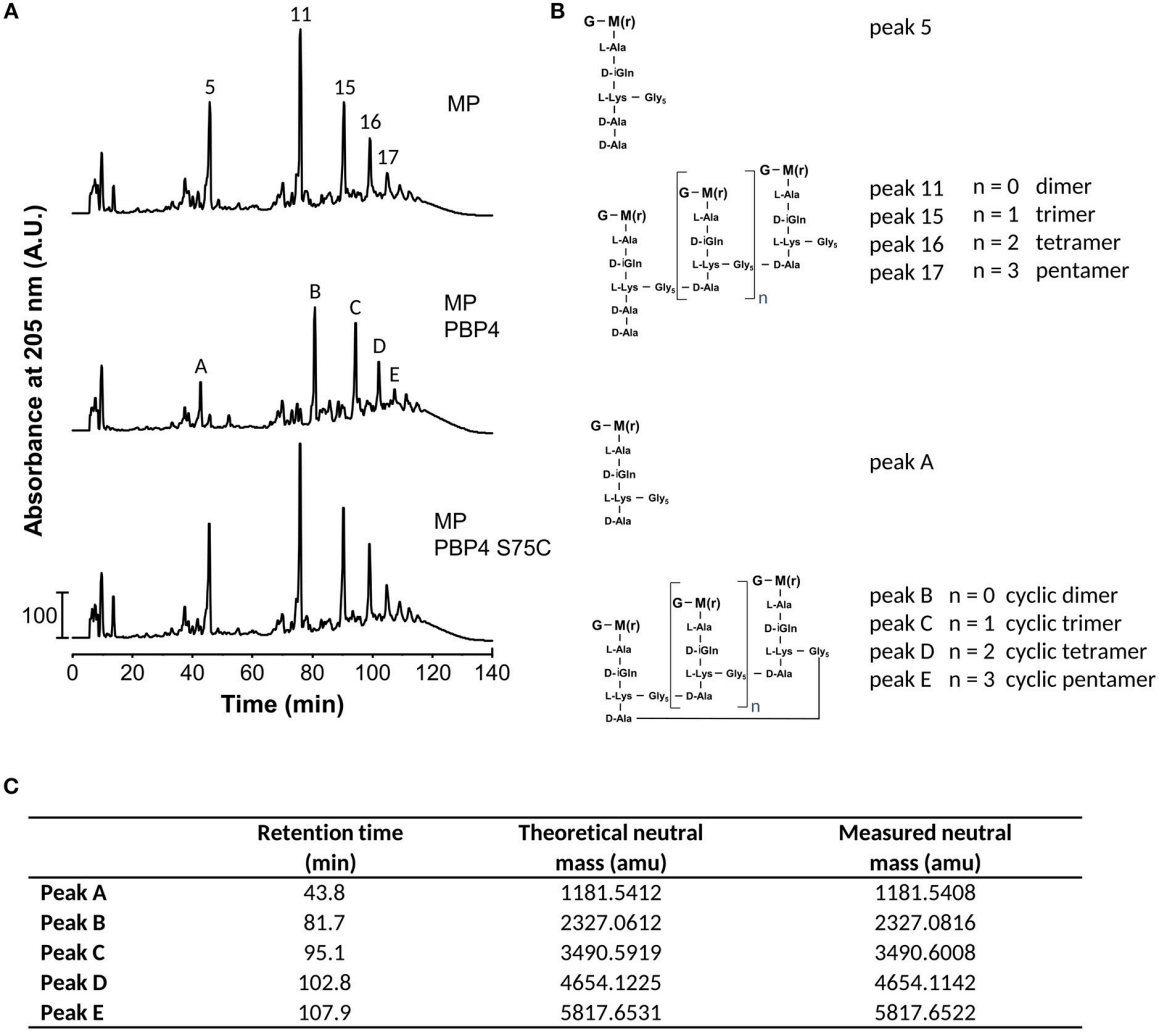
PBP4 forms cyclic muropeptides *in vitro*. **(A)** HPLC chromatograms of muropeptides from *S. aureus* SH1000 incubated with PBP4 or the inactive version PBP4(S75C). **(B)** Proposed structures of muropeptides present in the fractions in panel A and consistent with the molecular weight determined by mass spectrometry. G, *N*-acetylglucosamine; M(r), *N*-acetylmuramitol; L-Ala, L-alanine; D-iGln, D-isoglutamine; D-Ala, D-alanine; L-Lys, L-lysine; Gly, glycine. **(C)** Theoretical and measured neutral atomic mass units (amu) determined by MS of fractions collected from the PBP4 products in the middle chromatogram in **(A)**.

To test the activity of *S. aureus* PBP4, we incubated the enzyme with muropeptides from *S. aureus* SH1000 and analyzed the reaction products by HPLC and MS (Figure 3). PBP4 showed DD-CPase activity against the monomeric muropeptide, disaccharide pentapeptide(Gly_5_) (peak5), which was nearly quantitatively converted to disaccharide tetrapeptide(Gly_5_) (peak A). Interestingly, all the oligomeric peaks (for example, the dimer 11, trimer 15, tetramer 16, or pentamer 17) were completely converted by PBP4 to products with higher retention times (peaks B, C, D, and E). MS analysis of these products identified these as cyclic products, ostensibly as a result of intramolecular TPase activity of PBP4 (Figures 3B,C). As expected, the active site mutant PBP4(S75C) was inactive in this assay (Figure 3A). Remarkably, although PBP4(S75C) lacked CPase or TPase activity with muropeptide substrates, this version was nevertheless sensitive to acylation by β-lactams and, more specifically, by the carbapenem class compound imipenem as shown by MS (Supplementary Figure S3).

### PBP4 Does Not Interact With Synthetic Stem Peptides

X-ray structures of wild-type PBP4 alone or in complex with β-lactams have been previously deposited in the PDB (PDB IDs: 6C39, 5TW8, 5TXI, 5TY7). Here we chose to study interactions with stem peptides using the catalytic serine mutant (S75C) to prevent any possible TPase activity that might interfere with the formation of a complex amenable for structural characterization by X-ray crystallography or NMR spectroscopy.

PBP4(S75C) was crystallized under similar conditions to those previously used for wild-type PBP4 (Alexander et al., 2018), yielding data to 1.86-Å resolution. Supplementary Table 2 shows the details of the data collection and refinement statistics for PBP4(S75C). PBP4(S75C) crystallized with two highly similar molecules present in the asymmetric unit (Cα alignment of chains A and B gives an r.m.s.d. of 0.35 Å over 359 aligned atoms). As expected, the structure of PBP4(S75C) (deposited under PDB ID 6DZ8) closely aligns with the structure of wild-type PBP4 (PDB ID 6C39), giving a Cα r.m.s.d. of 0.41 Å over 359 aligned atoms (Supplementary Figure S4). Despite soaking ampicillin and the synthetic pentapeptide stem with PBP4(S75C) or PBP4, we were unable to identify electron density for either of these compounds in the maps generated.

**Figure 4:**
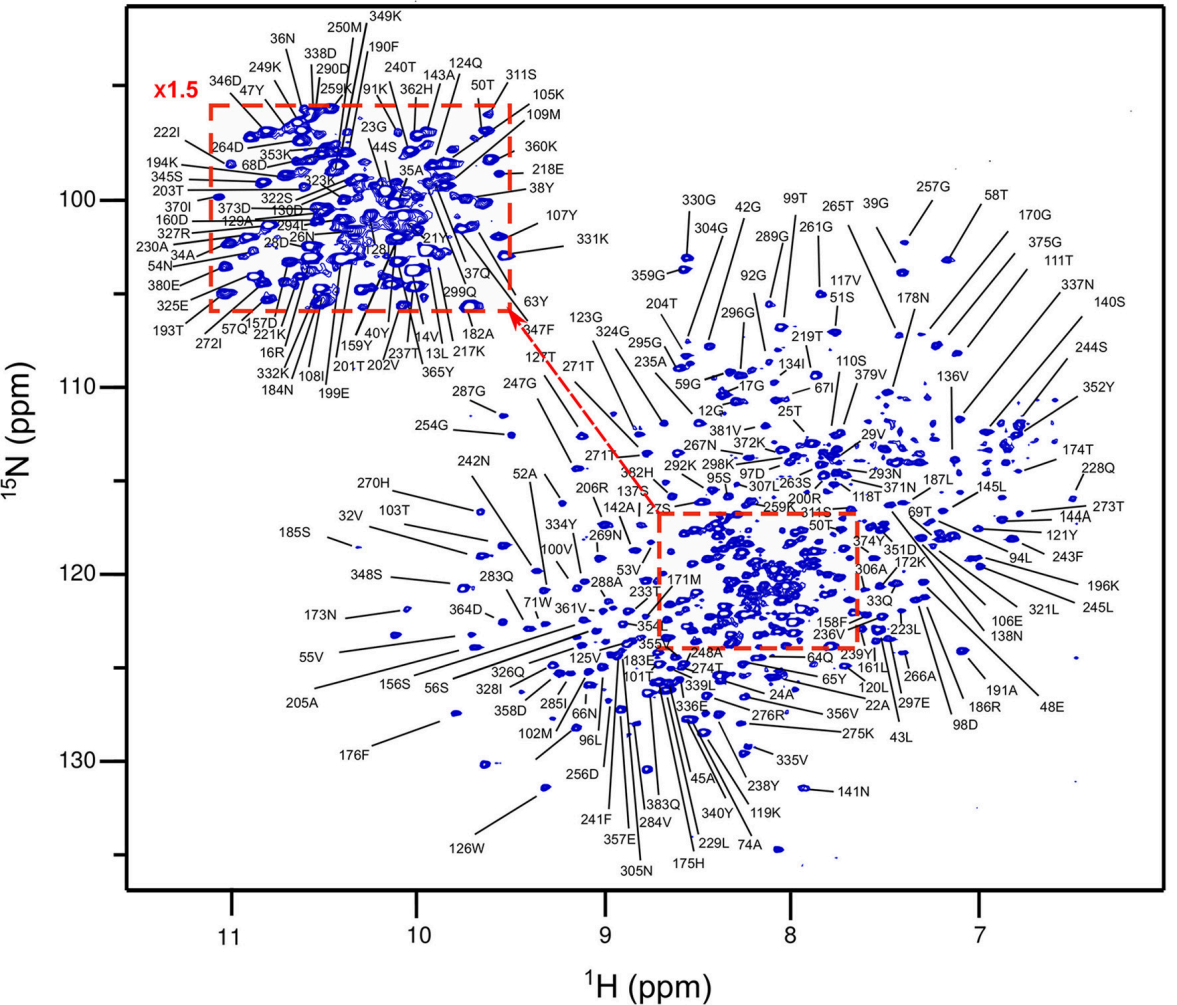
Assignment of the amide resonances in PBP4. Display of the ^1^H,^15^N-BEST-TROSY spectrum of a PBP4 sample (800 μM) in 100 mM potassium phosphate buffer containing 300 mM KCl and 5% D_2_O at pH 7.5, collected at 25°C on an 850-MHz spectrometer. Assignment of backbone amide resonances obtained from 3D experiments is reported. Only 92 over 321 detected amide resonances remained unassigned due to the low sensitivity in 3D experiments.

In the absence of a clear extra density that could correspond to the peptide stem in the PBP4 or PBP4(S75C) crystals, we investigated this interaction by liquid-state NMR. Assignment of the sequential backbone resonances of PBP4 was first achieved by using a series of conventional 3D heteronuclear experiments recorded on a ^1^H,^13^C,^15^N-labeled sample of PBP4 complemented with data on a ^2^H,^13^C,^15^N-labeled sample due to the size (over 30-kDa) of the protein (Figure 4). Backbone resonance assignment was achieved at 70% and was mainly limited by sensitivity issues in the 3D experiments in relation to the relatively high molecular weight of the protein. Assignments were transferred to PBP4(S75C) by superimposing the 2D ^1^H,^15^N-BEST-TROSY experiments (Favier and Brutscher, 2011) of the two proteins (Supplementary Figure S5). The spectra showed a very limited number of chemical shift variations, indicating that the structure is not significantly affected by the S75C substitution. This is in agreement with the highly similar structures of the wild-type and mutated protein determined by X-ray crystallography at similarly high resolution.

**Figure 5:**
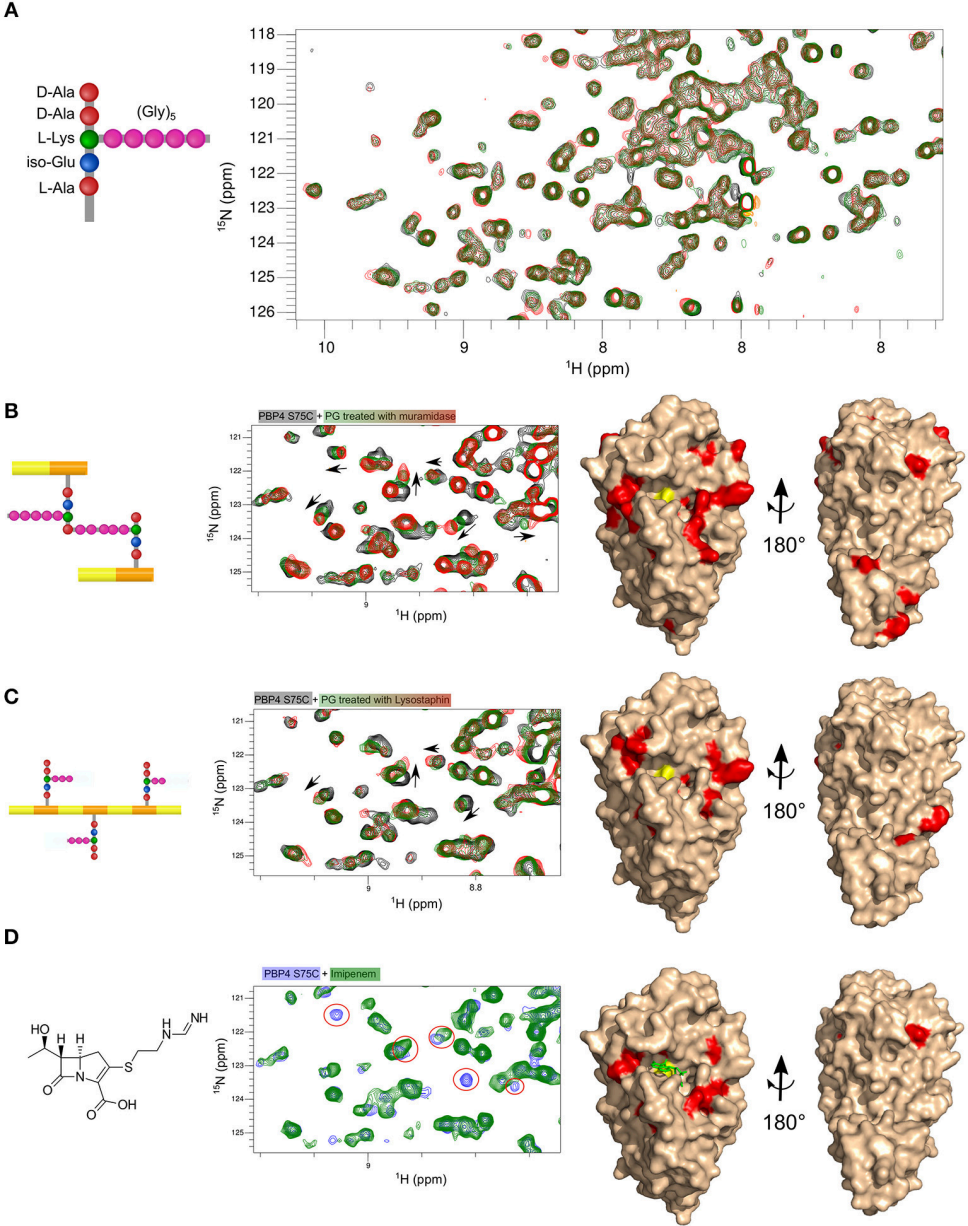
Mapping the interaction surface of PBP4(S75C) with synthetic peptides, PG fragments and imipenem. Parts of ^1^H,^15^N-BEST-TROSY spectra of PBP4(S57C) before and after addition of different ligands which are shown on the left side: **(A)** lactoyltetrapeptide HO-D-Lac-L-Ala-D-iGln-[L-Lys(Gly)_5_]-D-Ala-COOH (black, green, and red with 1:0, 1:22, and 1:52 protein:peptide molar ratios, respectively, at a protein concentration of 326 μM); **(B)** muropeptides from *S. aureus* strain SH1000 (black, green, and red with 1:0, 1:50, and 1:180 protein:fragment molar ratios for a protein concentration of 150 μM) **(C)** PG fragments obtained by digestion with lysostaphin (black, green, and red with 1:0, 1:10, and 1:16 protein:fragment molar ratios for a protein concentration of 150 μM); **(D)** imipenem (blue and green with 1:0 and 1:1.2 protein:antibiotic molar ratios for a protein concentration of 150 μM). Right panels in **(B–D)** display a surface representation of the PBP4 structure in beige. Residues showing significant chemical shift perturbations after addition of ligands at the higher ligand-to-protein ratio are color-coded in red. The active-site serine is shown in yellow.

A 326 μM sample of ^15^N-labeled PBP4(S75C) was then incubated with increasing amounts (2, 8, 22, and 52 molar equivalents to the protein) of the synthetic unlabeled lactoyl-tetrapeptide HO-D-Lac-L-Ala-D-iGln-L-Lys(Gly)_5_-D-Ala-COOH, and 2D ^1^H,^15^N-correlation spectra (^15^N-BEST-TROSY) were collected for each titration point (Figure 5A). The addition of the peptide did not create any significant chemical shift perturbations for the amide resonances of PBP4(S75C), showing that any possible interaction of the stem peptide with the protein must have a dissociation constant that exceeds 110 mM. This result likely explains the failure to obtain co-crystals or loaded (soaked) crystals of PBP4(S75C) and PBP4 with synthetic peptides.

### PBP4 Interacts With Larger PG Fragments

We asked whether the low affinity of PBP4 for small peptide substrates, too low for structural studies by either X-ray or NMR, could be due to the absence of the GlcNAc-MurNAc disaccharide motif or extended glycan strands. To address this question, we tested for interactions between PBP4(S75C) and PG fragments obtained by either cellosyl or lysostaphin digestion, using the same NMR approach as before (Figures 5B,C, left). In both cases, the ^1^H,^15^N-correlation spectra revealed small but consistent chemical shift perturbations following the addition of increasing amounts of PG fragments, suggesting binding with a fast exchange regime and a dissociation constant in the range of hundreds of μM to a few mM.

Our next goal was to determine the interaction site(s) on PBP4(S75C) for the soluble peptidoglycan fragments. Chemical shift perturbations were calculated for the highest fragment-to-protein ratio of 180 and 16 molar equivalents for the soluble fragments obtained by cellosyl and lysostaphin digestion (Supplementary Figure S6), respectively, and reported on the PBP4 structure to localize interaction interfaces on the protein (Figures 5B,C, right). These results were compared to the one obtained for the interaction of PBP4(S75C) with the previously mentioned carbapenem, imipenem (Figure 5D). We found that the natural substrates and the antibiotic bound to the same regions of PBP4 that are shared with the published binding sites of cephems (ceftaroline) and penams (nafcillin) on wild-type PBP4. However, we observed additional residues with perturbed chemical shifts in the case of the soluble PG fragments, suggesting a more extended interface encompassing surfaces flanking both sides of the central serine nucleophile and catalytic pocket. The positioning of these two extended surfaces suggests that they indeed mimic the binding sites of the extended donor and acceptor substrates of the TPase reaction. Several residues remote from the β-lactam binding site and located in the C-terminal domain of PBP4 (I328, K331, V336, F347, V356, and V361) also experienced significant chemical shift perturbations with muropeptides (Figure 5, Supplementary Figure S7). These shifts could be caused by a secondary muropeptide binding site or a remote allostery.

### Structural Model of a PBP4:Muropeptide Complex

**Figure 6:**
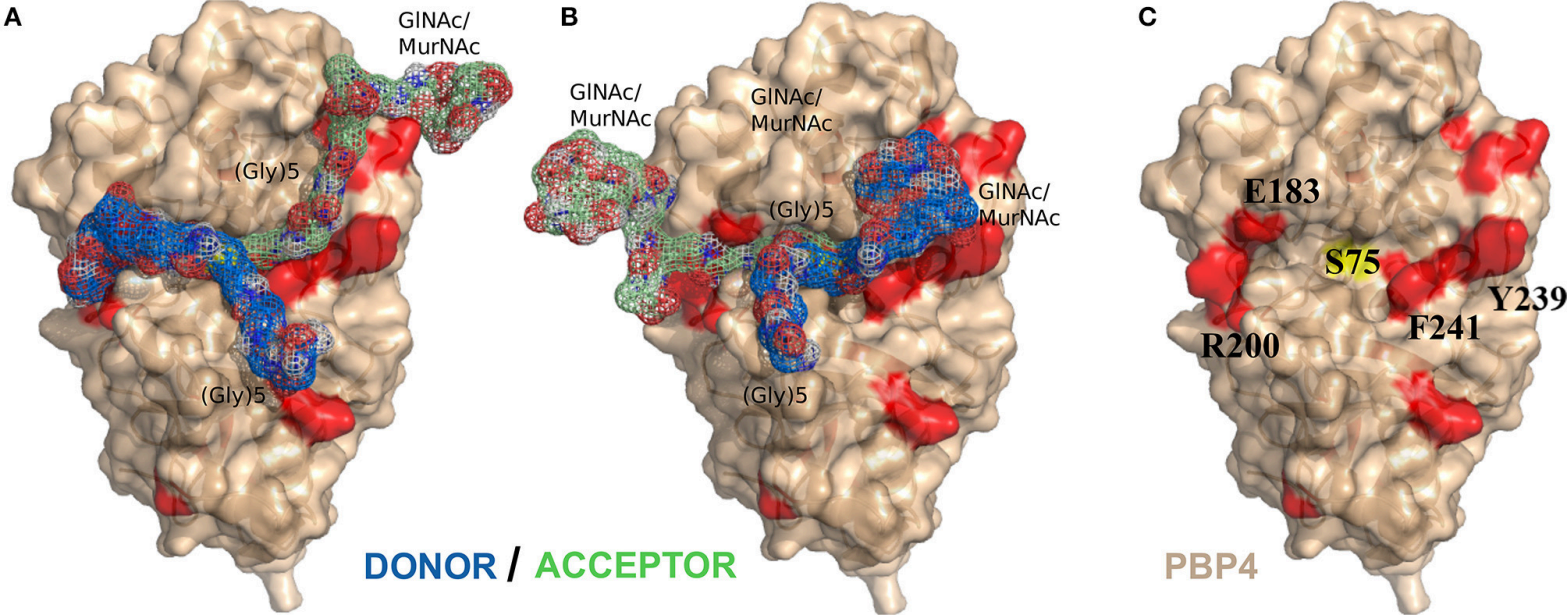
NMR-data driven model of the PBP4:muropeptide complex. **(A)** Lowest energy structure of the optimal cluster solution obtained with HADDOCK (Haddock score of 93.5 ± 2.7). **(B)** Lowest energy structure of the second optimal cluster (Haddock score of −93.3 ± 3.7). Donor and acceptor muropeptide substrates are colored in blue and green, respectively. **(C)** Surface representation of the structure of PBP4 in beige. Residues of PBP4 with significant NMR chemical shift perturbations and active site serine are colored in red and yellow, respectively. The residues highlighted show a side-chain reorientation between the PBP4 protein alone and in complex with muropeptides.

We then calculated a model of the complex formed between PBP4 and the acceptor and donor muropeptides using HADDOCK 2.2/CNS by docking two muropeptide structures with tetrapeptide stems onto the PBP4 structure and using the measured NMR chemical shift perturbations as ambiguous distance restraints to drive the docking during the energy minimization process. Based on the evidence that S75 is required for the TPase catalysis (by analogy to the reactivity with carbapenem antibiotic), the distances from the S75 serine oxygen atom to both the carbonyl carbon of D-Ala^4^ of the donor peptide stem and the amine nitrogen atom at the N-terminus of the pentaglycine bridge of the acceptor peptide were constrained to 2.5 Å. These two restraints, justified by the necessity to bind both substrate peptides near the active site residue, improved the convergence of this multi-body docking process. The calculation was run with 4,000 structures during the rigid body energy minimization and 200 structures during the first and second energy refinements (the latter in explicit water). All residues of the protein that made intermolecular contacts within a 5-Å cutoff in the initial rigid body docking were allowed structural reorientations during the simulated annealing process. The muropeptides were assumed fully flexible and all of their atoms were considered as ambiguous interaction sites to facilitate the sampling of different structural conformations at all steps of the simulated annealing process. The final minimization of the complexes in water revealed the presence of two clusters of similar energies (HADDOCK score: −93.5 ± 2.7 and −93.3 ± 3.7; electrostatic energy: −375.5 ± 70.0 and −416.2 ± 5.5 kcal mol^−1^; desolvation energy: −10.9 ± 10.1 and −0.1 ± 4.1 kcal mol^−1^; cluster size 120 and 35 structures, respectively) (Figures 6A,B). They show an inverse position of the donor and acceptor muropeptides relative to the catalytic pocket (centered on the central nucleophile S75 as depicted in Figure 6C).

## Discussion

*S. aureus* PBP4 is an unusual class C PBP showing TPase activity *in vivo* (Loskill et al., 2014; Hamilton et al., 2017) and *in vitro* (Qiao et al., 2014; Srisuknimit et al., 2017) in addition to its expected CPase activity. PBP4 is involved in β-lactam resistance in the *S. aureus* laboratory mutant 27s, in which the deletion of PBP4 led to a decrease in cross-linking and an increase in ceftizoxime susceptibility. The authors further observed that deletion of the PBP4 gene resulted in the disappearance of a peak within the muropeptide profile (Leski and Tomasz, 2005), which was previously identified as a cyclic muropeptide predominantly naturally occurring in methicillin- and cefotaxime-resistant mutants of *S. aureus* (Boneca et al., 1997). However, there was no direct evidence for the hypothesis that PBP4 produces these cyclic structures. Here we show that purified PBP4 is indeed able to catalyze the formation of cyclic muropeptides *in vitro*. The cyclisation reaction might merely be the result of a hyper-active TPase enzyme. Alternatively, cyclisation reactions might have the function to limit the availability of free PG strands to undergo intermolecular cross-linking. Furthermore, cyclic muropeptides might be a poorer substrate for PG hydrolases (autolysins) and might thus protect the cell from lysis due to an imbalance of synthetic and hydrolytic enzymes. Testing these hypotheses should provide new insights into the mechanism by which PBP4 is capable of conferring *mecA*-independent resistance to β-lactams in *S. aureus*.

Investigating the mechanism of transpeptidation by PBP4 with atomistic details requires different substrates or substrate mimetics in quantities amenable for structural biology methods. Here we developed the synthesis of branched lactoyl peptides using orthogonal protecting groups, which could be completed in future work with the anchoring of disaccharides or longer glycan chains to the lactoyl moiety. We have also isolated PG fragments from *S. aureus* cells and characterized their interaction with PBP4 by NMR spectroscopy, which is particularly useful to analyze mixtures without further purification and decrease in overall yields (Figure 2D).

Our different crystallization or soaking assays of PBP4, in the presence of synthetic pentapeptide or tetrapeptide stems or soluble peptidoglycan fragments obtained by enzymatic digestion, point out the difficulty in obtaining a high-resolution crystallographic structure of PBP4 in complex with mimetic or natural substrates. This difficulty may be explained by the low affinity of PBP4 for its substrate, the partial instability of the donor/PBP4 acylenzyme and/or the flexibility and heterogeneity of the soluble fragments obtained by digestion. NMR is an alternative technique to screen for interaction with a specific substrate among heterogeneous mixtures and to extract structural information on complexes with relatively weak affinities (Carlomagno, 2012). We therefore measured chemical shift perturbations induced by the substrates onto the enzyme in order to probe for interactions. Our NMR data show that a simple peptide stem does not yield to significant complex formation, while a complete muropeptide permits the detection of significant chemical shift perturbations near the catalytic pocket. We used these NMR data as experimental constraints to dock the muropeptide on PBP4, and the initial structures were energy minimized. We obtained models for the complex that satisfied the experimental observations of two different interaction regions for the disaccharide-peptide units, which could represent the donor and acceptor strands (Figures 6A,B). In the two final models, the donor and acceptor peptides access the catalytic pocket from two opposite sites, thus solving the steric problem of approaching the two Lys and (Gly)_5_ bulky chains near the catalytic serine S75. The somewhat similar energies obtained for the two clusters did not permit however to discriminate unequivocally between the two sets of solutions. In the lowest energy model (Figure 6A), the donor strand follows an orientation that would place the D-Ala^4^-D-Ala^5^ scissile bond in a similar geometry to that of the opened β-lactam ring in the acylenzyme (PDB IDs 5TW8, 5TXI, 5TY7). The lysine side-chain and glycine bridge then point in the same direction as the β-lactam's adjacent ring side-chain, while the terminal nitrogen of the pentaglycine bridge of the acceptor strand lines up with the β-lactam nitrogen. This arrangement is consistent with the analogy model suggested by Tipper and Strominger in 1965 for the donor substrate and the inhibitor (Tipper and Strominger, 1965). In the second model (Figure 6B), the donor stem approaches the catalytic cavity on its more open side (F241, Y239) and the terminal carboxylic group of the D-Ala localizes at the same place as the β-lactam carboxylate in the structure of the acylenzyme (PDB IDs 5TW8, 5TXI, 5TY7). In this model, the longest and narrowest side of the catalytic pocket (E183, R200) is occupied by the long glycine chain of the acceptor (Figure 6B). This model can explain the selectivity of PBP4 during the transpeptidation reaction for acceptor peptide stands containing complete (Gly_5_) chains (Srisuknimit et al., 2017). Both models suggest that side-chain reorientations must occur in particular for residues R200, E183, R186, E114, and F241 during the docking protocol in order to accommodate the substrates (Supplementary Figure S8A). This structural reorganization enhances accessibility of the long peptide stems to the otherwise more closed S75 catalytic cavity (Figure 6C).

Interestingly, a ceftobiprole-resistant *Staphylococcus aureus* strain (CRB), which displays resistance to a variety of β-lactams, has three mutations in the *pbp4* gene that result in E183A and F241R amino-acid substitutions in PBP4 (Banerjee et al., 2010). The structure of the latter protein acylated by the ceftobiprole and ceftaroline cephems has been solved by crystallography (PDB IDs 5TX9, 5TW4) and shows few structural differences in proximity to the antibiotic (Alexander et al., 2018). The replacement of E183 by an alanine disrupts the Van-der-Waals interaction between this residue and R200 and induces a rotation of R200, increasing significantly the accessibility of the catalytic pocket for one of the substrates, donor and acceptor peptide stems in models of Figures 6A,B, respectively (Supplementary Figure S8B). The mutation of F241 to an arginine could have a similar impact on the accessibility of the catalytic serine S75 to the second substrate, acceptor and donor stem peptide models of Figures 6A,B, respectively. Based on our results, we suggest that the CRB mutations, by changing the accessibility to the catalytic serine S75, facilitates the transpeptidation of the two substrates, whereas this could also facilitate the hydrolysis of the antibiotic acylenzyme. These hypotheses need to be further tested *in vitro* and *in vivo* in CRB mutants.

## Author Contributions

RM-M contributed to NMR sample preparation and was in charge of NMR data collection and analysis on PBP4 and PBP4(S75C). JA was in charge of all of the X-ray PBP4(S75C) sample preparation, data collection and analysis. CO was in charge of the characterization of the peptidoglycan. IA has prepared all peptidoglycan and most protein samples for NMR. DV has prepared all peptidoglycan samples for HPLC and mass spectrometry. JG was in charge of the mass spectrometry. CB has performed some NMR data collection and analysis and has contributed to MS writing. CL was in charge of the docking with HADDOCK. AB has assigned the NMR peptidoglycan fragments and participated in PBP4-fragment interaction studies. MF has synthesized the tetra- and pentapetide stems. MA has supervised the production of the peptide stems. NS has supervised the X-ray. WV has supervised the peptidoglycan characterization and has contributed to the MS writing. J-PS has supervised the complete study and has contributed to MS writing and supervision.

### Conflict of Interest Statement

The authors declare that the research was conducted in the absence of any commercial or financial relationships that could be construed as a potential conflict of interest.
