# Supplementary material for: Recognition of Peptidoglycan Fragments by the Transpeptidase PBP4 From Staphylococcus aureus
Source: Front Microbiol. 2019 Jan 18;9:3223. doi: 10.3389/fmicb.2018.03223 (PMC6346638; doi:10.3389/fmicb.2018.03223)
Supplement: Supplementary file 1 [file Data_Sheet_1.PDF]

SUPPLEMENTARY INFORMATION

Recognition of peptidoglycan fragments by the transpeptidase PBP4 from *Staphylococcus aureus* Recognition of peptidoglycan fragments by the *Staphylococcus aureus* PBP4 transpeptidase

Roberto Maya-Martinez [1], J. Andrew N. Alexander [2], Christian Otten [3,#], Isabel Ayala [1], Daniela Vollmer [3], Joe Gray [4], Catherine M. Bougault [1], Alister Burt [1], Cédric Laguri [1], Matthieu Fonvielle [5], Michel Arthur [5], Natalie C.J. Strynadka [2], Waldemar Vollmer [3] and Jean-Pierre Simorre [1,*]

[1] Univ. Grenoble Alpes, CNRS, CEA, IBS, F-38000 Grenoble, France

[2] Department of Biochemistry and Molecular Biology and Centre for Blood Research, The University of British Columbia, Vancouver V6T 1Z3, British Columbia, Canada

[3] Centre for Bacterial Cell Biology, Institute for Cell and Molecular Biosciences, Newcastle University, Richardson Road, Newcastle upon Tyne, NE2 4AX, Unighted Kingdom

[4] Institute for Cell and Molecular Biosciences, Newcastle University, Framlington Place, Newcatsle upon Tyne, NE1 7RU, United Kingdom

[5] Centre de Recherche des Cordeliers, LRMA, Equipe 12, Université Sorbone–Paris, France

Table of contents

Supplementary material and methods 3

Supplementary Figures 4

Supp. Figure S1: 2D spectra for the assignment of synthetic tetrapeptide and the peptidoglycan fragments obtained by lysostaphin digestion. 4

Supp. Figure S2: HPLC and MS quality control of the synthetic peptide stems. 5

Supp. Figure S3: Mass spectra of PBP4(S75C) before and after incubation with imipenem. 6

Supp. Figure S4: Alignment of PBP4(S75C) and PBP4 X-ray structures. 7

Supp. Figure S5: Superimposition of the ^{15}N-BEST-TROSY spectra of PBP4 and PBP4(S75C). 8

Supp. Figure S6: Chemical shift perturbations induced on PBP4(S75C) by the binding of different substrates. 9

Supp. Figure S7: Mapping of the chemical shift perturbations induced on PBP4(S75C) by the binding of different substrates. 10

Supp. Figure S8: Superimposition of 4 structures of PBP4 and PBP4(E183A/F241R) 11

Supplementary Tables 12

Supp. Table 1: ^{1}H- and ^{13}C-resonance assignment of synthetic tetra- and penta-peptide. 12

Supp. Table 2: Data collection and structure refinement statistics for PBP4(S75C). 13

Supplementary material and methods

Analysis of branched lactoyl peptides in complement to main text and reference Ngadjeua et al., 2018. After isolation of the unprotected tetrapeptide or pentapeptide material, the crude product was dissolved in 10% acetic acid (5 mL) and extracted 3 times with 10 mL of chloroform. The aqueous fraction was lyophilised. The peptides were then dissolved in water and purified by *rp*HPLC on a 5-μm Nucleosil preparative C-18 column (22 × 250 mm) using a linear gradient from 0 to 100% of 0.1% TFA buffer in CH_3CN, applied between 10 and 40 minutes at a flow rate of 10 mL min^{-1}, from a starting equilibration condition of the column in 0.1% TFA in H_2O. Peptide-containing fractions were identified at 214 nm and isolated. Corresponding fractions were lyophilised and dissolved in water at a final concentration of approximately 10 mg mL^{-1}. Peptide concentration was determined in duplicate by acid hydrolysis and injection into a Hitachi L8800 amino acid analyser equipped with a 2620 MSC-PS column. Purity was assessed by *rp*HPLC (analytical C18 Nucleosil column, 3 μm, 4.6 x 250 mm) using a linear gradient (0 to 20% buffer B) applied between 5 and 20 min (buffer A: 0.1 % TFA in H_2O; buffer B: 0.1 % TFA in CH_3CN; 2 mL min^{-1}) and chemical structure was confirmed by mass spectrometry (Supplementary Figure S2), and NMR (Supplementary Table 1).

Supplementary Figure S1

(A)

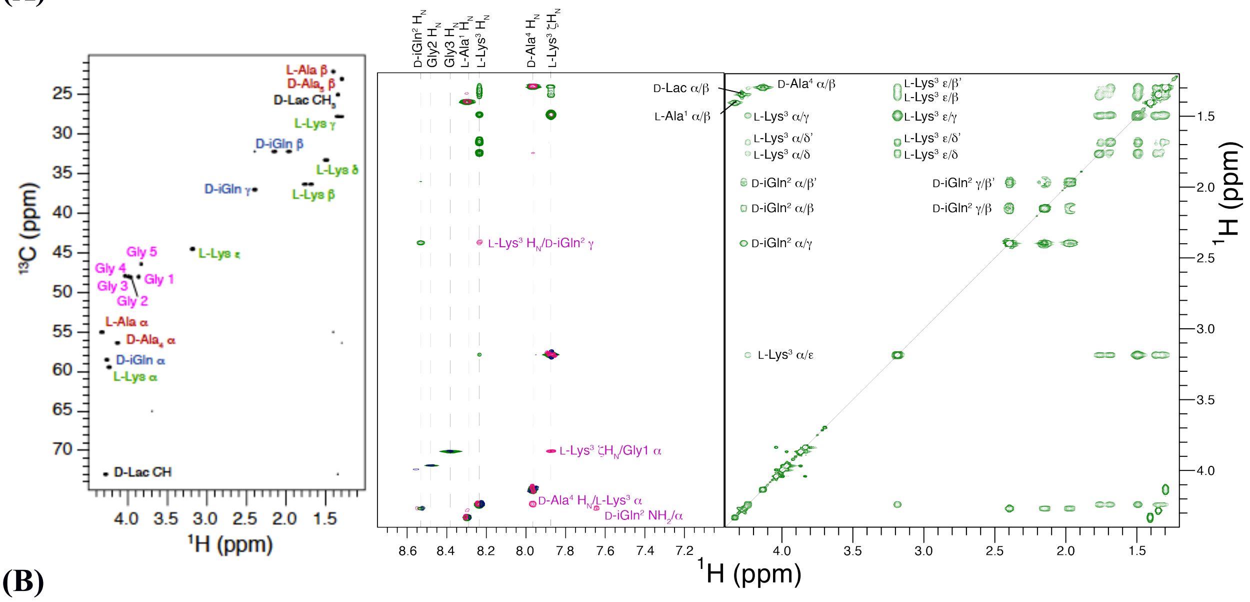

(B)

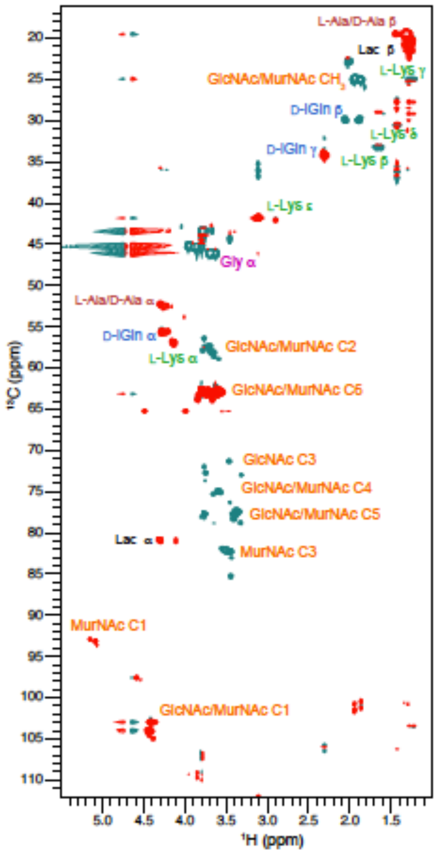

Supplementary Figure S1: 2D spectra for the assignment of synthetic tetrapeptide and the peptidoglycan fragments obtained by lysostaphin digestion. (A) $^{1}H,^{13}C$-HSQC (left) and superimposition of the [$^{1}H,^{1}H$]-TOCSY and [$^{1}H,^{1}H$]-NOESY in green and pink, respectively, (right) collected at 20 °C on a 2 mg mL^{-1} sample of HO-D-Lac-L-Ala-D-iGln-L-Lys(Gly)$_5$-D-Ala-COOH tetrapeptide in 50 mM potassium phosphate buffer at pH 6.5 containing 10% D_2O. The aliphatic region of the TOCSY on the right allows identification of the amino acid type, while the amide region of the NOESY on the left allows specific residue assignment through the H_N-to-Hα correlations. (B) [$^{1}H,^{13}C$]-HSQC spectrum collected at 20 °C of a ~1 mM solution of $^{13}C,^{15}N$-labelled peptidoglycan fragments obtained by lysostaphin digestion in 50 mM potassium phosphate buffer at pH 6.5 containing 10% D_2O.

Supplementary Figure S2

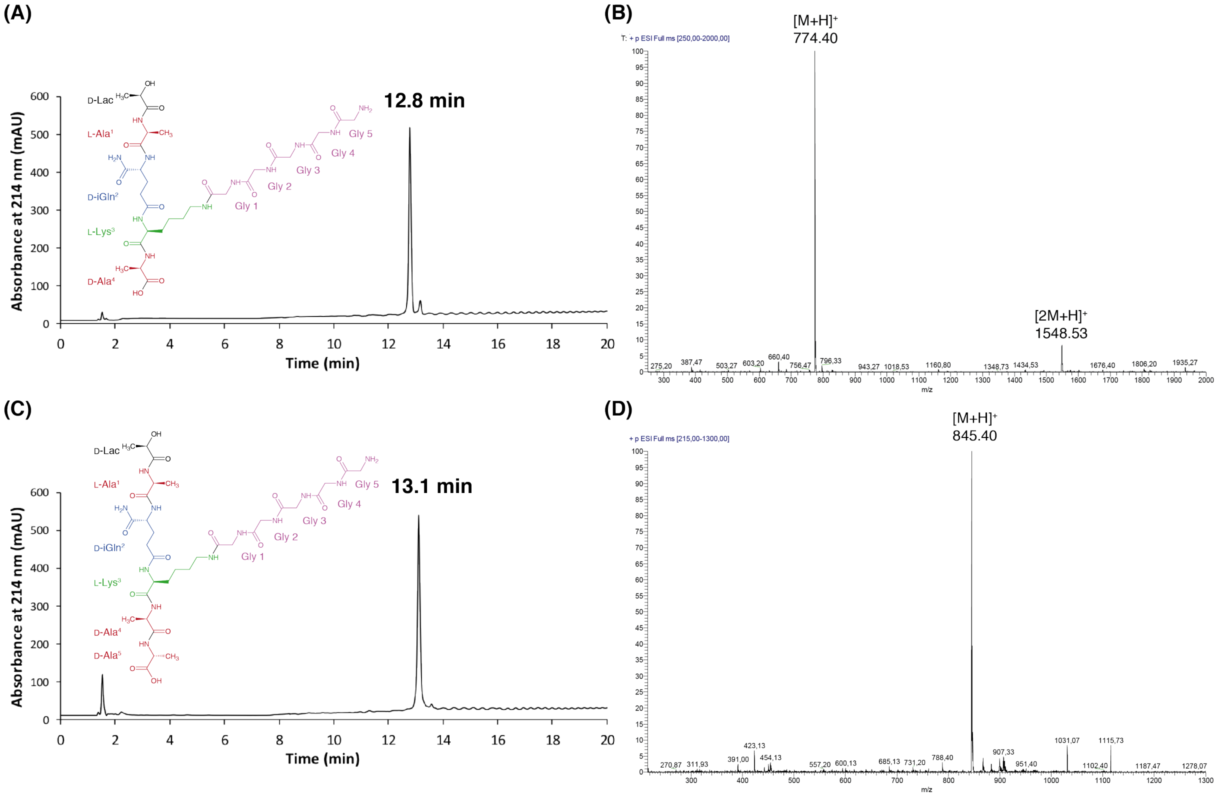

Supplementary Figure S2: HPLC and MS quality control of the synthetic peptide stems. (A,C) HPLC on a 3 µm analytical C18 Nucleosil column (4.6 × 250 mm) using a linear gradient (0 to 20% buffer B) applied between 5 and 20 min (buffer A: 0.1 % TFA in H_2O; buffer B: 0.1 % TFA in CH_3CN; 2 mL min^{-1}). (B,D) High-resolution mass spectra (MS) recorded on a TOF mass analyser under electrospray ionization conditions. Injected samples are 10 mg mL^{-1} tetrapeptide (A,B) or pentapeptide (C,D) solutions in water.

Supplementary Figure S3

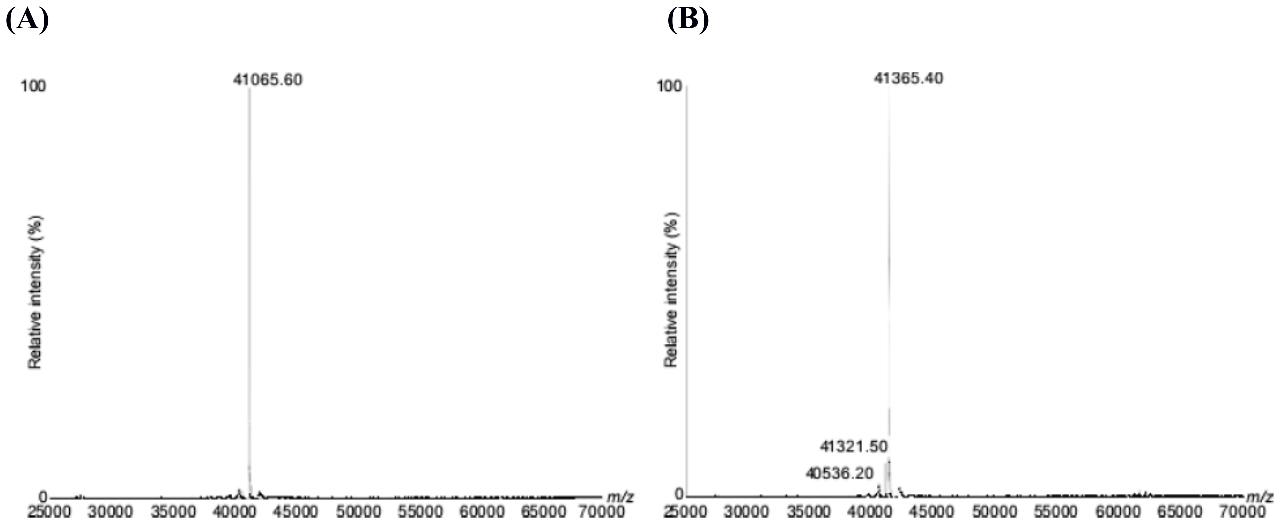

Supplementary Figure S3: Mass spectra of PBP4(S75C) before and after incubation with imipenem. The detected molecular masses of 41,065.60 Da and 41,365.40 Da for the PBP4(S75C) sample before (A) and after (B) incubation with imipenem are highly consistent with the expected masses of 41,065.5 Da and 41,364.8 for PBP4(S75C) and PBP4(S75C) acylated by imipenem, respectively.

Supplementary Figure S4

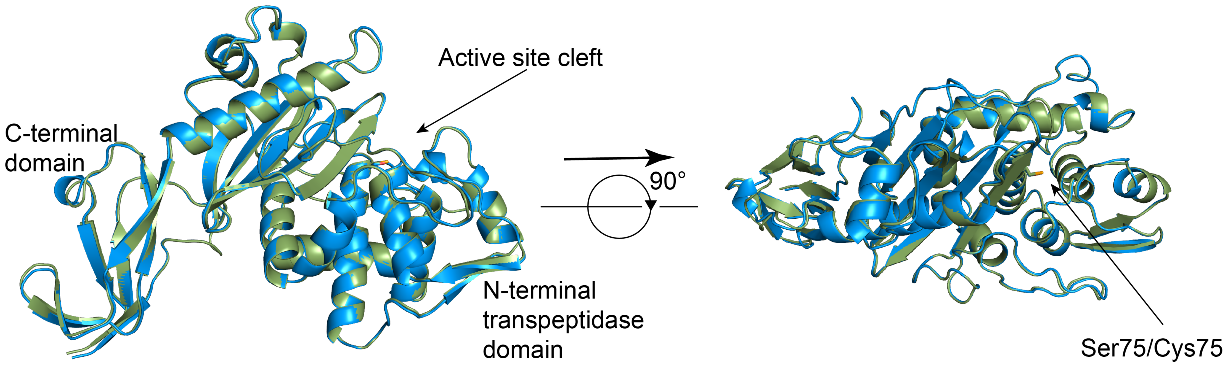

Supplementary Figure S4: Alignment of PBP4(S75C) (PDB ID 6DZ8) and PBP4 (PDB ID 6C39) X-ray structures. Both structures are depicted as cartoons with PBP(S75C) shown in green and PBP4 shown in blue. The catalytic serine S75 or C75 (in the case of PBP4(S75C)) are shown as sticks. Cα alignment of these two structures gives an r.m.s.d of 0.41 Å over 359 atoms.

Supplementary Figure S5

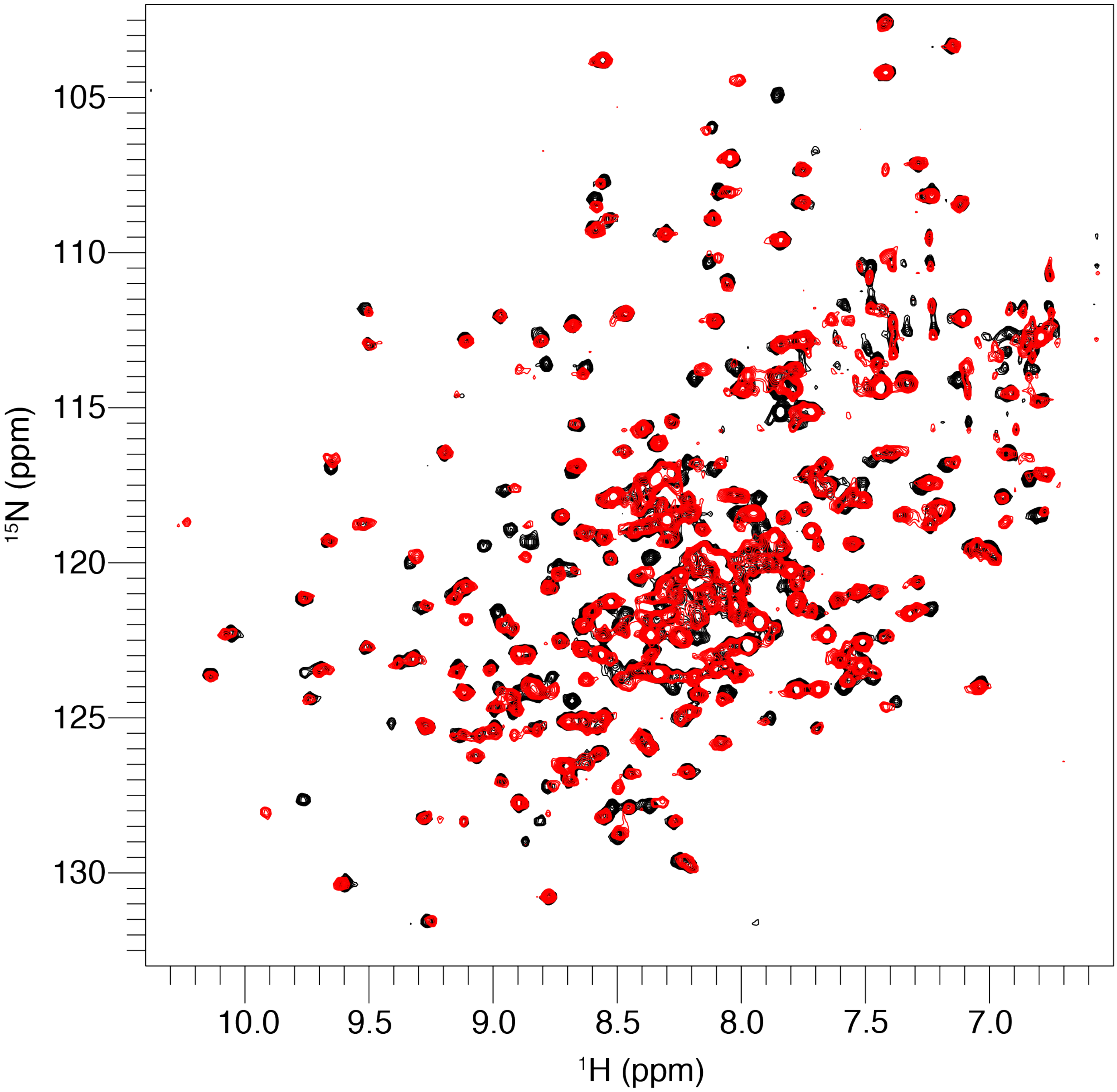

Supplementary Figure S5: Superimposition of the ^{15}N-BEST-TROSY spectra of PBP4 (black) and PBP4(S75C) (red) in 50 mM potassium phosphate buffer at pH 6.5 and 25 °C. The overlay of most resonances for the ^{15}N-only labelled samples indicates a preservation of the structure through the S75C replacement, in complete consistency with X-ray results. Changes are only located in the vicinity of residue S75. This superimposition allowed transferring assignment of amide resonances from the wild-type to the mutant protein.

Supplementary Figure S6

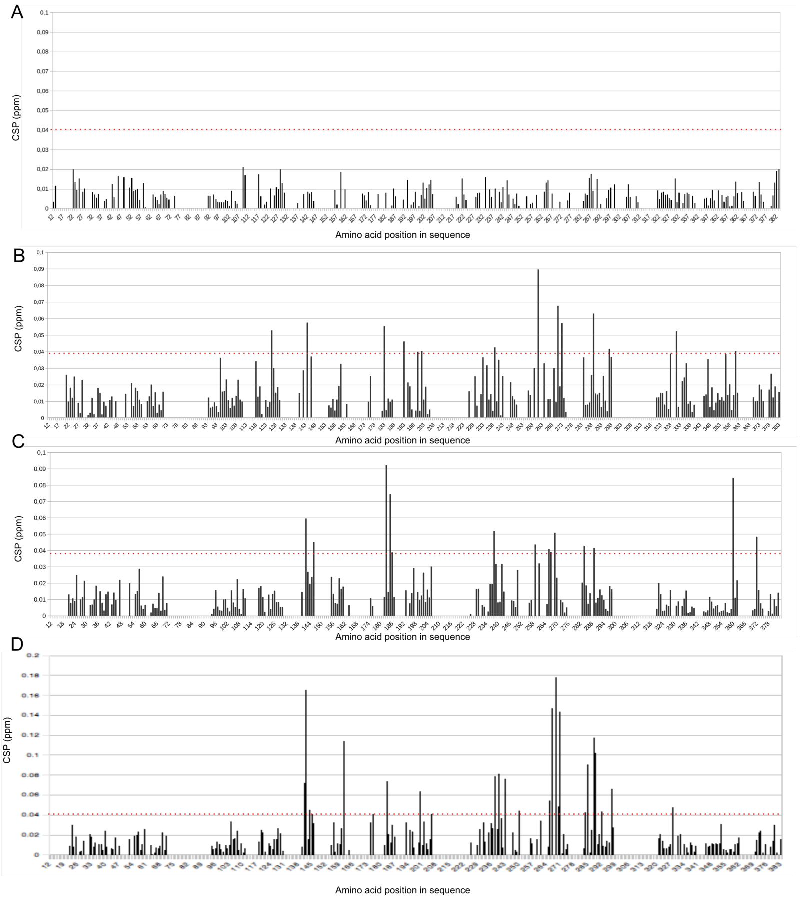

Supplementary Figure S6: Chemical shift perturbations induced on PBP4(S75C) by the binding of different substrates. The following substrates were added at 25 °C on 150-µM PBP4(S75C) solution in 50 mM potassium phosphate buffer, pH 6.5: (A) Tetrapeptide at approximately 52 molar equivalents to the protein, (B) Muropeptides from *S. aureus* strain SH1000 at approximately 50 molar equivalents to the protein, (C) Peptidoglycan fragments obtained after lysostaphin digestion at 15-20 molar equivalents to the protein, and (D) Imipenem at 1.2 molar equivalents to the protein. Imipenem binds covalently to PBP4^{S75C}, to form a stable acylenzyme as detected in mass spectrometry. The red line indicates the threshold (average CSP value + 2 standard deviations) above which CSPs are considered significant, and is used to report affected residues on the protein structure. Data points where there is no visible histogram correspond to unassigned or severely overlapped residues, for which CSPs could not be determined.

Supplementary Figure S7

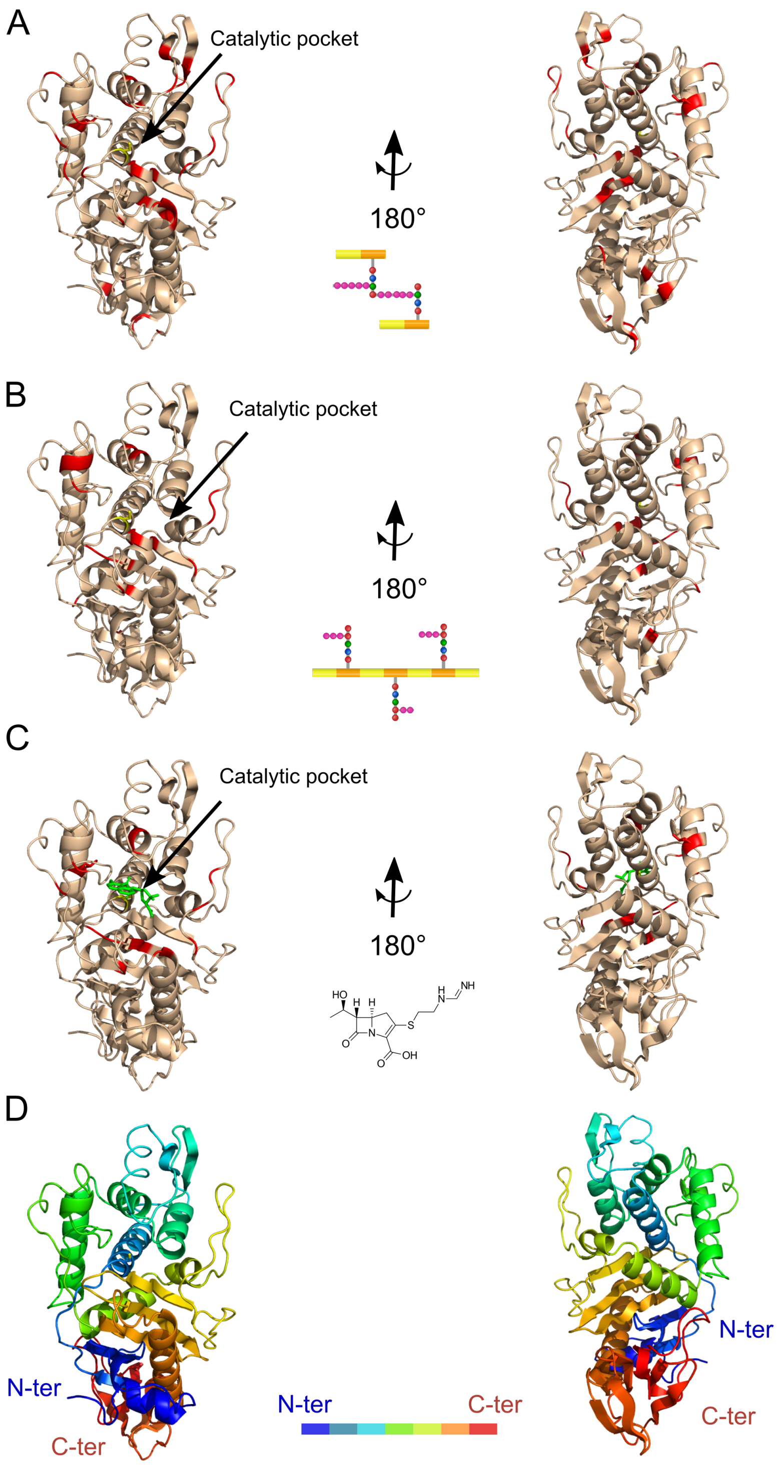

Supplementary Figure S7: Residues with significant chemical shift perturbations in Supplementary Figure S6 are colour-coded in red on a ribbon representation of PBP4 (A) using muropeptides from *S. aureus* strain SH1000, (B) using peptidoglycan fragments obtained after lysostaphin digestion, (C) using imipenem. (D) Ribbon representation of PBP4 coloured from the N-terminus to the C-terminus using a colour gradient from blue to red.

Supplementary Figure S8

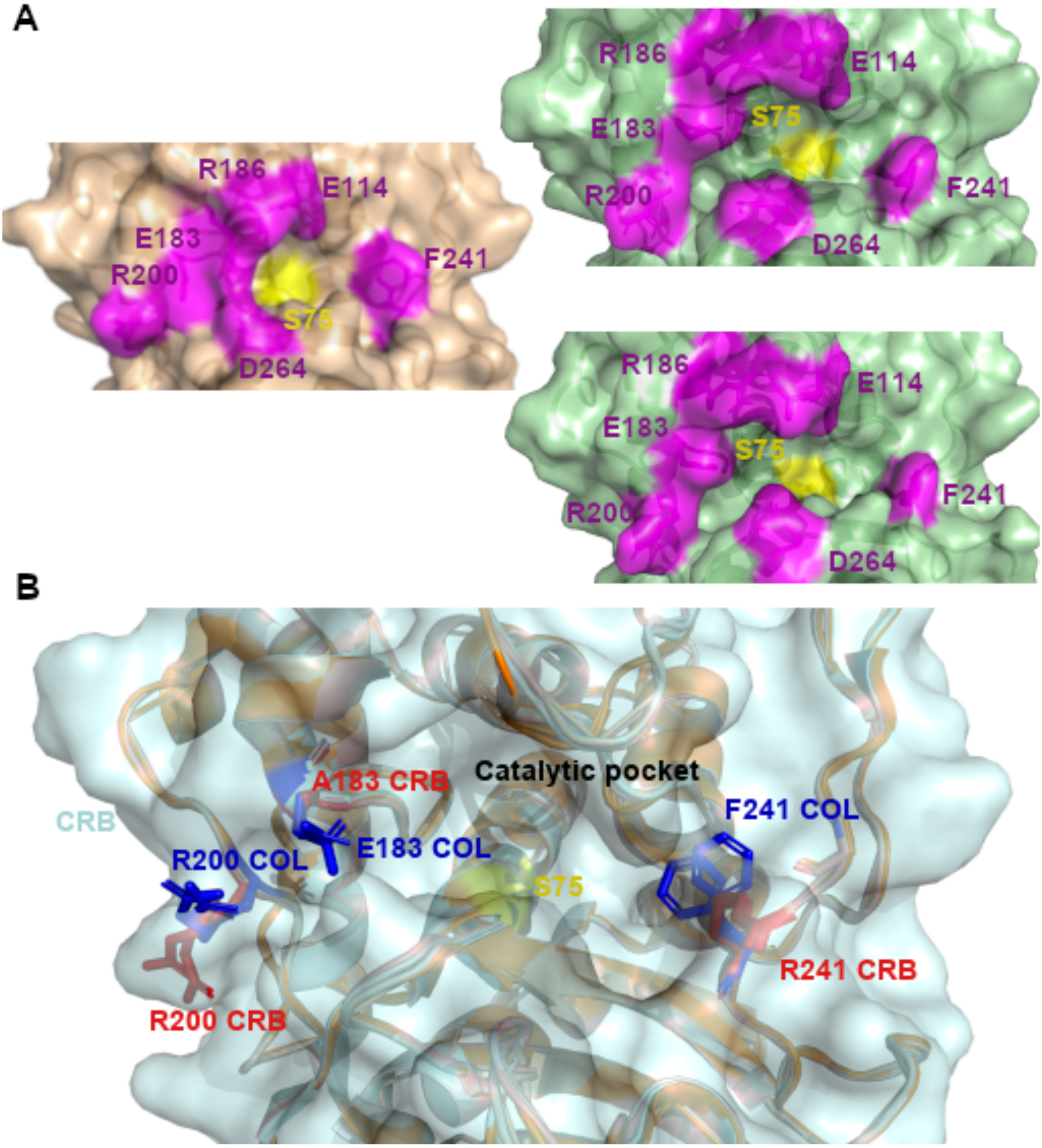

Supplementary Figure S8: Accessibility of the catalytic pocket (A) for the structure of the PBP4 COL apo-form (left panel), and for the two final clusters minimized in the presence of muropeptides (right panel). Residues showing the larger side-chain reorientation during the docking process are coloured in purple, while the catalytic S75 is coloured in yellow. (B) Superimposition of 4 PDB structures of the wild-type PBP4 (PBP4 COL) and the double PBP4 mutant E183A/F241R (PBP4 CRB) resistant to ceftobiprole. The PBP4 CRB in the presence of ceftaroline (PDB ID 5TW4), PBP4 COL in the presence of ceftaroline (PDB ID 5TW8), PBP4 CRB in the presence of ceftobiprole (PDB ID 5TX9), and PBP4 COL in the presence of ceftobiprole (PDB ID 5TXI) structures are shown as ribbons in wheat, orange, pink, and pale gray, respectively. A surface representation of the 5TW4 structure is also shown in pale cyan. Residues R200, E183, A183, F241, or R241 that show evidence for side-chain reorientations are shown as sticks on the respective structures.

Supplementary Table 1

Residue	^{1}H nuclei							^{13}C nuclei				
	H_N	Hα	Hβ	Hγ	Hδ	Hε	NH_{sc} or NH_{2sc}	Cα	Cβ	Cγ	Cδ	Cε
	**Tetrapeptide chemical shifts (ppm)**											
D-Lac		4.269	1.348					73.01	25.01			
L-Ala^{1}	8.298	4.331	1.408					55.03	22.05			
D-iGln^{2}	8.531	4.269	1.966<br>2.150	2.396			7.315<br>7.646	58.52	32.17	36.99		
L-Lys^{3}	8.235	4.240	1.688<br>1.763	1.299<br>1.348	1.492	3.186	7.875	59.46	36.31	27.74	33.24	44.50
D-Ala^{4}	7.964	4.141	1.299					56.40	22.99			
Gly 1	8.381	3.868						48.03				
Gly 2	8.476	3.966						48.08				
Gly 3		3.997						47.99				
Gly 4		4.042						47.90				
Gly 5		3.837						46.40				
	**Pentapeptide chemical shifts (ppm)**											
D-Lac		4.269	1.334					73.01	24.98			
L-Ala^{1}	8.298	4.321	1.394					55.04	22.05			
D-iGln^{2}	8.521	4.251	1.953<br>2.129	2.373			7.129<br>7.642	58.48	32.12	36.84		
L-Lys^{3}	8.239	4.183	1.684<br>1.732	1.284<br>1.337	1.484	3.173		59.63	36.01	27.71	33.25	44.48
D-Ala^{4}	8.355	4.330	1.339					54.88	22.06			
D-Ala^{5}	7.921	4.067	1.303					56.54	22.74			
Gly 1	8.364	3.855						48.02				
Gly 2	8.463	3.954						48.06				
Gly 3	8.541	3.982						47.97				
Gly 4		4.031						47.88				
Gly 5		3.857						46.16				

Supplementary Table 1: ^{1}H- and ^{13}C-resonance assignment of synthetic HO-D-Lac-L-Ala-D-iGln-L-Lys(Gly)$_5$-D-Ala-COOH tetrapeptide and the HO-D-Lac-L-Ala-D-iGln-[L-Lys(Gly)$_5$]-D-Ala-D-Ala-COOH pentapeptide in 50 mM potassium phosphate buffer with 10% D_2O at pH 6.5 and 20 °C (see Figure 2B in main text for residue numbering)

Supplementary Table 2

**Data collection**	
Beamline	ALS 5.0.2
Space group	C121
Cell dimensions	
a, *b*, *c* (Å)	104.5, 89.7, 77.5
α, β, γ, (°)	90.0, 98.1, 90.0
Wavelength (Å)	1.0000
Resolution (Å)*	28.73 – 1.86 (1.93 – 1.86)
Number of unique reflections	59156 (5860)
R_{merge}	0.097 (1.032)
CC1/2	0.997 (0.566)
I/σ*I*	8.46 (1.31)
Completeness (%)	99.4 (99.3)
Multiplicity	3.4 (3.3)
**Refinement**	
Resolution (Å)	28.73 – 1.86
$R_{work/}$ R_{free}	0.175/0.216
Number of non-hydrogen atoms	
Protein	5662
Ion	4
Water	303
B-factors (Å^2)	
Protein	32.1
Ion	47.1
Water	37.6
r.m.s.d.	
Bond lengths (Å)	0.008
Bond angles (º)	1.16
Ramachandran favored/allowed/disallowed (%)	98.3, 1.7, 0.0

Data corresponds to diffraction from a single crystal. Coordinates are deposited in the Protein Data Bank under the accession number 6DZ8.

* Highest resolution shell is shown in parenthesis.

Supplementary Table 2: Data collection and structure refinement statistics for PBP4(S75C)
